# MRI visual rating scales in the diagnosis of dementia: evaluation in 184 post-mortem confirmed cases

**DOI:** 10.1093/brain/aww005

**Published:** 2016-03-01

**Authors:** Lorna Harper, Giorgio G. Fumagalli, Frederik Barkhof, Philip Scheltens, John T. O’Brien, Femke Bouwman, Emma J. Burton, Jonathan D. Rohrer, Nick C. Fox, Gerard R. Ridgway, Jonathan M. Schott

**Affiliations:** ^1^ Dementia Research Centre, University College London Institute of Neurology, London WC1N 3BG, UK; ^2^ Neurology Unit, Department of Physiopathology and Transplantation, University of Milan, Fondazione Cà Granda, IRCCS Ospedale Policlinico, Milan, Italy; ^3^ Department of Radiology and Nuclear Medicine, VU University Medical Centre, 1081 HZ Amsterdam, The Netherlands; ^4^ Alzheimer Centre, VU University Medical Centre, 1081 HZ Amsterdam, The Netherlands; ^5^ Department of Psychiatry, University of Cambridge, Cambridge CB2 0SZ, UK; ^6^ Institute for Ageing and Health, Newcastle University, Campus for Ageing and Vitality, Newcastle upon Tyne NE4 5PL, UK; ^7^ FMRIB Centre, Nuffield Department of Clinical Neurosciences, University of Oxford OX3 9DU, UK; ^8^ Wellcome Trust Centre for Neuroimaging, UCL Institute of Neurology, London WC1N 3BG, UK

**Keywords:** brain atrophy, dementia, MRI, neuropathology, visual rating

## Abstract

Accurately distinguishing between different degenerative dementias during life is challenging but increasingly important with the prospect of disease-modifying therapies. Molecular biomarkers of dementia pathology are becoming available, but are not widely used in clinical practice. Conversely, structural neuroimaging is recommended in the evaluation of cognitive impairment. Visual assessment remains the primary method of scan interpretation, but in the absence of a structured approach, diagnostically relevant information may be under-utilized. This definitive, multi-centre study uses post-mortem confirmed cases as the gold standard to: (i) assess the reliability of six visual rating scales; (ii) determine their associated pattern of atrophy; (iii) compare their diagnostic value with expert scan assessment; and (iv) assess the accuracy of a machine learning approach based on multiple rating scales to predict underlying pathology. The study includes T _1_ -weighted images acquired in three European centres from 184 individuals with histopathologically confirmed dementia (101 patients with Alzheimer’s disease, 28 patients with dementia with Lewy bodies, 55 patients with frontotemporal lobar degeneration), and scans from 73 healthy controls. Six visual rating scales (medial temporal, posterior, anterior temporal, orbito-frontal, anterior cingulate and fronto-insula) were applied to 257 scans (two raters), and to a subset of 80 scans (three raters). Six experts also provided a diagnosis based on unstructured assessment of the 80-scan subset. The reliability and time taken to apply each scale was evaluated. Voxel-based morphometry was used to explore the relationship between each rating scale and the pattern of grey matter volume loss. Additionally, the performance of each scale to predict dementia pathology both individually and in combination was evaluated using a support vector classifier, which was compared with expert scan assessment to estimate clinical value. Reliability of scan assessment was generally good (intraclass correlation coefficient > 0.7), and average time to apply all six scales was <3 min. There was a very close association between the pattern of grey matter loss and the regions of interest each scale was designed to assess. Using automated classification based on all six rating scales, the accuracy (estimated using the area under the receiver-operator curves) for distinguishing each pathological group from controls ranged from 0.86–0.97; and from one another, 0.75–0.92. These results were substantially better than the accuracy of any single scale, at least as good as expert reads, and comparable to previous studies using molecular biomarkers. Visual rating scores from magnetic resonance images routinely acquired as part of the investigation of dementias, offer a practical, inexpensive means of improving diagnostic accuracy.

## Teaser

Distinguishing between the degenerative dementias during life can be challenging. Using histopathologically confirmed cases for comparison, Harper *et al.* evaluate six visual rating scales for use with routinely acquired structural MRI. Automated classification based on all six scales provides a practical, fast and inexpensive means of improving diagnostic certainty.

## Graphical abstract

**Figure:**
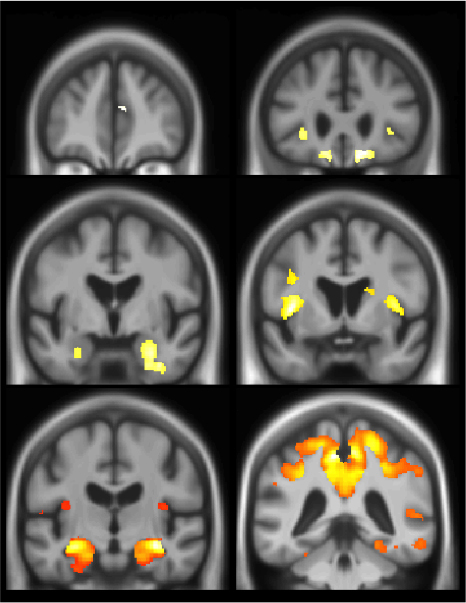
Distinguishing between the degenerative dementias during life can be challenging. Using histopathologically confirmed cases for comparison, Harper *et al.* evaluate six visual rating scales for use with routinely acquired structural MRI. Automated classification based on all six scales provides a practical, fast and inexpensive means of improving diagnostic certainty.

## Introduction

Distinguishing between the different neurodegenerative causes of dementia is vitally important to allow affected individuals and their families to access appropriate treatment, support and care ( Gaugler *et al.* , 2013 ). This requirement will become even more pressing as disease-modifying therapies become available. With the exception of rare autosomal dominant forms of dementia, accurate diagnosis during life can be challenging, as distinct underlying pathologies can result in overlapping clinical symptoms ( Schott and Warren, 2012 ). Post-mortem examination of brain tissue, therefore, currently remains the diagnostic gold standard ( Beach *et al.* , 2012 ). Pathologically, the degenerative dementias are linked by protein misfolding in the brain, with the specific abnormal protein and its pattern of deposition defining each neurodegenerative disease. These include the accumulation of hyperphosphorylated tau and extracellular deposition of amyloid-β in Alzheimer’s disease ( Hyman *et al.* , 2012 ); the aggregation of alpha-synuclein in dementia with Lewy bodies (DLB) ( McKeith *et al.* , 2005 ); and the accumulation of several proteins including 3-repeat and 4-repeat tau, and TAR DNA-binding protein 43 (TDP-43) in frototemporal lobar degeneration (FTLD) ( Mackenzie *et al.* , 2010 ). Whilst biomarkers of the molecular pathology of Alzheimer’s disease, including CSF analysis of amyloid-β, tau and phosphorylated tau ( Ewers *et al.* , 2015 ), or amyloid PET ( Jack *et al.* , 2013 ), are available in some expert centres, logistical challenges and financial constraints limit their adoption into routine clinical use at this time. By contrast, structural neuroimaging is widely available and recommended as part of the clinical evaluation in all patients with suspected dementia [ National Collaborating Centre for Mental Health (UK), 2006 ] and in the diagnostic criteria for a number of different dementias ( Hort *et al.* , 2010 ; Gorno-Tempini *et al.* , 2011 ; Rascovsky *et al.* , 2011 ). The high resolution and excellent tissue contrast afforded by MRI in particular allows for global and regional cerebral atrophy to be assessed, offering positive predictive value for underlying disease pathology ( Harper *et al.* , 2014 ).

While a number of sophisticated methods of analysis are available to quantify global and regional atrophy from MRI ( Dale *et al.* , 1999 ; Leung *et al.* , 2011 ; Cardoso *et al.* , 2013 ; Schrouff *et al.* , 2013 ), relatively little progress has been made to integrate these into clinical work streams due to special hardware requirements, prohibitively long processing times and dependency on specific acquisition techniques. Accordingly, visual scan assessment remains the primary method for extracting diagnostically useful information in clinical settings. However, without operational guidelines to identify, report or interpret patterns of atrophy with diagnostic value in dementia, much potentially relevant information may be under-utilized. Visual rating scales, specifically designed to assess general and focal cerebral atrophy in patients with cognitive impairment (reviewed in Harper *et al.* , 2015 ), may provide such a framework, allowing for the reliable identification and interpretation of imaging findings of value in the differential diagnosis of dementia. Furthermore, since visual rating scales are both quick and easy to apply, and can be performed on routinely acquired images, they offer an inexpensive means of extracting this information, ideally suited for implementation into clinical practice, and may make it easier for clinicians without expertise in neuroradiology to extract diagnostically useful information.

Several visual rating scales have been developed specifically to rate brain regions vulnerable to atrophy in a range of different dementias. While some have been used extensively in both research and clinical settings, most notably the Scheltens’ medial temporal lobe scale ( Scheltens *et al.* , 1992 ), many have only been evaluated in small single centre studies. Few studies have attempted to compare directly or to combine the diagnostic value of individual scales. Fewer still have used a large multi-centre setting to determine the real-world generalizability and robustness of such findings, and to our knowledge, no study has exclusively assessed their diagnostic utility when applied to scans acquired from individuals with pathologically confirmed dementias. Using structural magnetic resonance scans from healthy individuals and a large sample of patients with a histopathological diagnosis of dementia, this study: (i) evaluates the reliability of six different visual rating scales and the time taken to perform these ratings; (ii) explores the relationship between each visual rating scale and the pattern of grey matter volume loss; (iii) compares the performance of rating scales to expert scan assessment in predicting underlying pathology; and (iv) determines whether a machine learning (support vector) approach, based on all visual rating scale scores, can improve prediction accuracy.

## Materials and methods

### Study population

Patients were identified who had a diagnosis of dementia during life and post-mortem ( *n* = 177) or a biopsy confirmation of the underlying pathology ( *n* = 7). Of these, 101 patients had a primary pathology diagnosis of Alzheimer’s disease [73 early-onset (<65 years at symptom onset), 28 late-onset (≥65 years at symptom onset)], 28 patients were diagnosed with DLB, and 55 with FTLD (24 tauopathies, 28 TDP-43 proteinopathies and three with fused-in sarcoma proteins). Pathological examination of brain tissue was carried out between 1997 and 2013 according to standard histopathological processes and criteria in use at the time of assessment at one of four centres: (i) the Queen Square Brain Bank, London; (ii) Kings College Hospital, London; (iii) VU Medical Centre, Amsterdam; and (iv) the Institute for Ageing and Health, Newcastle. Cognitively normal control subjects ( *n* = 73) were also included in the study. Ethical approval for the study was obtained from the National Research Ethics Service Committee London – South East.

### Structural MRI

All individuals had T _1_ -weighted volumetric MRI performed during life. As the data were collected retrospectively from multiple centres, the images were acquired on scanners from three different manufacturers (Philips, GE, Siemens) using a variety of different imaging protocols. Magnetic field strength varied between 1.0 T ( *n* = 21 scans), 1.5 T ( *n* = 204 scans) and 3.0 T ( *n* = 32 scans). For assessments, images were viewed using the in-house MIDAS (Medical Image Display and Analysis Software) image viewer ( Freeborough *et al.* , 1997 ), which allows for images to be viewed in axial, coronal and sagittal orientations, and for contrast and zoom to be altered.

### Visual rating of cerebral atrophy

**Figure 1:**
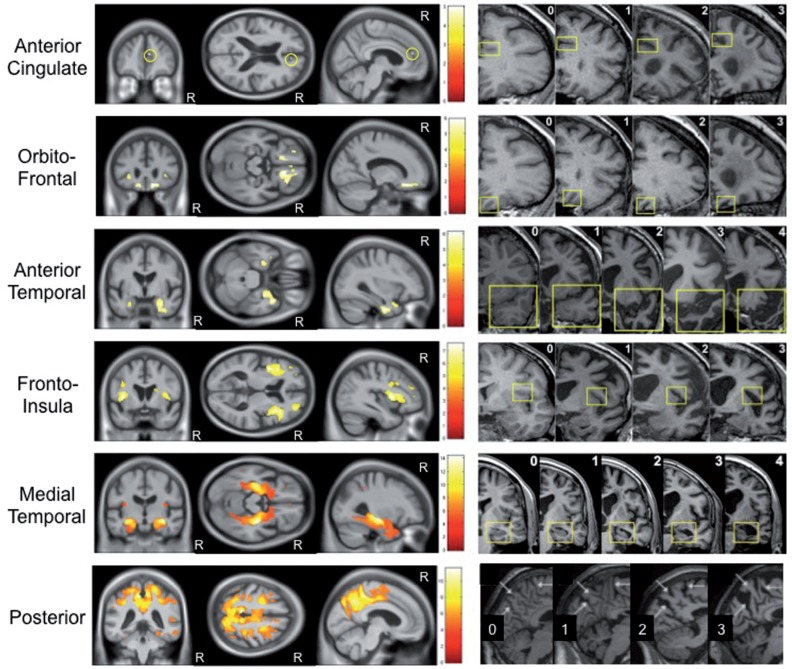
**Correlation between grey matter volume and visual rating score.** Voxel-based morphometry images demonstrating negative partial correlation between grey matter volume and each visual rating scale, adjusted for the other scales (Y = β _AC_ X _AC_ + β _OF_ X _OF_ + β _AT_ X _AT_ + β _FI_ X _FI_ + β _MTA_ X _MTA_ + β _PA_ X _PA_ + β _Age_ X _Age_ + β _Gender_ X _Gender_ + β _TIV_ X _TIV_ + β _1T_ X _1T_ + β _3T_ X _3T_ + β _London_ X _London_ + β _Amsterdam_ X _Amsterdam_ + μ + e). In all images statistical significance of correlations was corrected for multiple comparisons (family wise error rate *P* < 0.05). The corresponding visual rating scale reference images are displayed adjacent to each statistical parametric map. R indicates the right hemisphere.

Visual rating of the complete imaging dataset of all patients and controls ( *n* = 257) was performed, blind to all clinical and pathological information, by two trained raters (G.F. and L.H.). Three regions were rated based on existing scales previously described in the literature: (i) the five-point anterior temporal (AT) scale by Davies *et al.* (2006 ) and Kipps *et al.* (2007) ; (ii) the five-point medial temporal lobe atrophy (MTA) scale by Scheltens *et al.* (1992 ), previously recommended in the research guidelines for the diagnosis of Alzheimer’s disease ( Dubois *et al.* , 2007 ); and (iii) the four-point posterior atrophy (PA) scale by Koedam *et al.* (2011 ). To provide additional, more fine-grained assessment of anterior atrophy, we used an adapted and simplified version of a visual rating scale originally devised by Davies *et al.* (2009 ), as described by Fumagalli *et al.* (2014 ). In brief, three regions—orbito-frontal (OF), anterior cingulate (AC) and fronto-insula (FI)—previously shown to have potential for differential diagnosis ( Davies *et al.* , 2009 ; Hornberger *et al.* , 2010 ; Ambikairajah *et al.* , 2014 ) were selected. To improve usability, each scale was simplified to four points and reference images were devised. To improve consistency, slice selection was specified, with the OF and AC regions both rated on the first anterior slice where the corpus callosum becomes visible, and the FI rated over three slices, starting on the first anterior slice where the anterior cingulate becomes visible and moving posteriorly. Images were rated in native space, in keeping with standard clinical reads. To aid rating consistency, reference images for each rating scale were provided to the raters (examples provided in Fig. 1 and provided in full in the Supplementary material ). Separate scores were recorded for regions in left and right hemispheres. L.H. and G.F. initially performed visual rating training, applying the protocol described above to a sample of 150 images (50 controls, 100 with a clinical diagnosis of dementia) from research participants who attended the Dementia Research Centre, London.

To provide independent validation of the results from the two primary raters, two visual rating experts (F.B. and P.S.) also assessed 80 scans (20 Alzheimer’s disease, 20 DLB, 20 FTLD, 20 control) drawn at random from the total study population. G.F. also re-rated this subset population. The time taken by each rater to apply the visual rating protocol to each image was automatically recorded to estimate the feasibility of implementing such a protocol in clinical practice.

### Expert diagnosis

Six clinical dementia experts (F.B., N.F., J.O., P.S., J.S., G.F.), each provided what they thought was the most likely pathology diagnosis for the above-mentioned subset study population ( *n* = 80) based on independent, unstructured visual assessment of each MRI. Experts were blinded to all clinical and pathological information except the person’s age at the time of scanning. Images were displayed in a random sequence in terms of underlying pathology.

### Voxel-based morphometry

To explore the relationship between each rating scale and pattern of grey matter volume loss, voxel-based morphometry preprocessing and analysis was performed using SPM12b (Statistical Parametric Mapping, Version 12b revision 5829; http://www.fil.ion.ucl.ac.uk/spm ) and Matlab version R2012a (7.14.0.739 - 64-bit, uk.mathworks.com/products/matlab/). Due to the variability in scanning parameters, an initial rigid registration to the Montreal Neurological Institute International Consortium for Brain Mapping 152 (ICBM152) template was performed using the Reg-Aladin tool from the NiftyReg package ( Ourselin *et al.* , 2001 ; Modat *et al.* , 2010 ) to provide a better starting point for the statistical parametric mapping preprocessing pipeline. Each registration was then checked and manually adjusted (if necessary) such that the anterior commissure was within a few millimetres of the origin and the orientation was within a few degrees of the ICBM152 template. Grey matter, white matter and CSF were obtained using the unified segmentation approach ( Ashburner and Friston, 2005 ), which includes bias correction (regularization = 0.001, full-width at half-maximum = 60 mm) and rigid registration to the ICBM152 template. A group average tissue probability map was generated through iterative alignment of the initial segmentations to an evolving estimate of their group-wise average using the Dartel toolbox ( Ashburner, 2007 ; Ashburner and Friston, 2009 ). The initial grey and white matter segmentations were then warped using the Dartel transformations and modulated to account for local volume changes, then smoothed with a 6 mm full-width at half-maximum Gaussian kernel.

### Statistical analysis

Inter-rater reliability of each rating scale was determined using the intraclass correlation coefficient (ICC). As described by Shrout and Fleiss (1979) , there are several forms of ICC, with the appropriate form determined by the underlying statistical model and the intended application of the reliability results. In this study, a two-way random, absolute, single-measures ICC [ICC(2,1)] was used to estimate the reliability of each scale when applied by a single rater. ICC(2,1) was calculated separately for the subset group ( *n* = 80) based on four raters (F.B., G.F., P.S., L.H.), and the total study population ( *n* = 257) based on two raters (G.F., L.H.). Average measures ICCs [ICC(2,k)] were also calculated to estimate the improvement in reliability of each scale when based on average scores from multiple raters.

Partial correlation of grey matter volume with mean visual rating scores [based on the mean scores from four raters (F.B., G.F., L.H., P.S.) in the subset population and two raters (G.F., L.H.) in the remainder of the total population] was assessed by applying the general linear model at the level of each voxel using all images ( *n* = 257). Left and right hemisphere scores were averaged for each scale such that grey matter volume was modelled as a function of the six rating scales (OF, AC, AT, FI, MTA, PA) and adjusted for age, gender, total intracranial volume, magnetic field strength and acquisition site by including these variables as covariates in the model (equation included in Fig. 1 ). Six additional models were also created to investigate simple correlation of each individual scale with grey matter volume, including the covariates described in the larger model. A mask was created, based on the optimal threshold of the group average image, using the automatic mask creation strategy in the statistical parametric mapping toolbox ( Ridgway *et al.* , 2009 ). Correction for multiple comparisons was made by using random field theory to control the family-wise error rate at a significance level of 0.05.

Expert rater diagnosis was assessed for each binary disease group comparison and reported in terms of sensitivity, specificity and balanced accuracy [0.5 × (sensitivity + specificity)].

Analysis of the visual rating scale data, at the level of the individual scale and when the scales are combined in a linear support vector classifier, was based on the average scores across raters for each image in the study population (four rater average for the 80-scan subset, two rater average for the remaining scans). Left and right hemisphere scores were then averaged to create a mean score per visual rating scale (i.e. six scores per image), however, supplementary analysis was also performed based on the individual hemisphere scores (i.e. 12 scores per image) ( Supplementary Fig. 1 ). Group separation was investigated at the level of primary pathology group (Alzheimer’s disease, DLB, FTLD and control), and additionally at the subgroup level [early- (<65 years at symptom onset), and late-onset Alzheimer’s disease, DLB, FTLD-Tau, FTLD-TDP43, younger controls (<65 years at the time of scanning) and older controls].

The ability of each visual rating scale to predict pathology was assessed for each binary disease group comparison and reported in terms of sensitivity, specificity, balanced accuracy and area under the receiver-operator characteristic curve (AUC). Independent left and right hemisphere analysis was based on the highest of the two scores in all cases.

Separate linear support vector classifiers (SVC) were used to predict pathology for each binary comparison. Scores (features) were corrected for age at the time of scanning. Split-half separation was used to divide the data for each classifier into training and testing sets. The training data were scaled to zero mean and unit variance over subjects, with the same transformation then applied to the testing data. SVCs were trained using leave-one-out cross-validation on the training set. Class weighting was applied to adjust for unbalanced groups. The regularization parameter, C, was optimized using grid-search in the range 1 × 10 ^−5^ to 100, increasing by an order of magnitude each time. The SVC was implemented using the squared-hinge loss function and L2 regularization, with the algorithm set to solve the primal optimization problem. Classification accuracy is presented as balanced accuracy and receiver operator characteristic AUC values. Feature weighting for each classifier is discussed as an indication of each scale’s contribution to group separation ( Rakotomamonjy, 2003 ).

All data processing and analyses were performed using Python libraries NumPy 1.8.1 ( van der Walt *et al.* , 2011 ), SciPy 0.14.0 ( Jones *et al.* , 2001 ) and Pandas 0.14.1 ( McKinney, 2010 ) on Python 2.7.6 – 64-bit. SVC processing and analysis was performed using the Python libraries SciPy 0.14.0 ( Jones *et al.* , 2001 ) and Scikit-Learn 0.15.2 ( Pedregosa *et al.* , 2011 ). Confidence intervals were calculated using the Hanley-McNeil approach evaluated in Newcombe (2006) .

## Results

### Demographics

Demographic details of the patients and control subjects are shown in Table 1 . In terms of the primary groups (Alzheimer’s disease, DLB, FTLD and control), there were no significant differences between gender, disease duration and total intracranial volume. The patients with DLB were significantly older ( *P* < 0.001), with less time between scan until death ( *P* < 0.05) than the patients with Alzheimer’s disease and FTLD. The control subjects were also significantly older than the patients with Alzheimer’s disease or FTLD ( *P* < 0.05). Mini-Mental State Examination (MMSE) within 6 months of scan date was only available in 116 of the 184 patients (missing data in 37/101 Alzheimer’s disease, 5/28 DLB, 26/55 FTLD). Based on the data available, MMSE was significantly higher in the FTLD than the Alzheimer’s disease group ( *P* < 0.001). Similar results were found when using MMSE closest to scan ( *n* = 170/184).

**Table 1:** Patient demographics and mean visual rating scores

	**Controls**	**Younger controls**	**Older controls**	**Alzheimer’s disease**	**Early-onset Alzheimer’s disease**	**Late-onset Alzheimer’s disease**	**DLB**	**FTLD**	**FTLD-Tau**	**FTLD-TDP43**	**Significant differences**
*n*	73	33	40	101	73	28	28	55	24	28	NA
Gender (% male)	52%	70%	30%	61%	59%	68%	75%	56%	58%	50%	None
Age at scan (years)	66.6 (7.9)	59.9 (4.8)	72.2 (5.2)	61.1(11.4)	55.9 (8.1)	74.9 (5.5)	70.1 (5.9)	61.1 (8.8)	63.5 (8.5)	60.4 (8.3)	c, e, j * , l * , m * , n * , q * , r * , s, t * , u *
Disease duration at scan (years)	NA	NA	NA	3.7 (3.1)	4.1 (3.1)	2.8 (3.1)	3.0 (2.4)	3.3 (2.6)	3.9 (2.1)	3.0 (3.0)	None
Time from scan until death (years) ^a^	NA	NA	NA	5.6 (3.0)	5.4 (2.8)	5.9 (3.4)	3.5 (2.3)	5.3 (2.9)	5.0 (2.9)	5.6 (3.0)	j, l, m, p
MMSE within 6 months ^b^	NA	NA	NA	17.5 (6.0)	16.6 (6.3)	19.4 (4.8)	20.1 (4.6)	22.7 (5.9)	23.3 (5.2)	21.5 (6.8)	k * , n *
Total intracranial volume (ml)	1501 (159)	1442 (126)	1549 (168)	1479 (150)	1478 (158)	1482 (132)	1550 (148)	1498 (149)	1500 (149)	1474 (151)	None
**Mean visual rating scores**											
Orbito-frontal	0.9 (0.5)	0.8 (0.6)	1.0 (0.5)	1.6 (0.8)	1.5 (0.8)	1.7 (0.7)	1.5 (0.7)	2.3 (0.8)	2.3 (0.8)	2.2 (0.8)	c * , d * , e * , f * , g, h * , i * , k * , l * , n * , o * , q, s, t
Anterior cingulate	1.0 (0.5)	0.8 (0.5)	1.2 (0.5)	1.3 (0.7)	1.4 (0.7)	1.2 (0.6)	1.3 (0.5)	1.9 (0.8)	2.0 (0.9)	1.8 (0.7)	c, e * , f * , h * , i * , k * , l * , n * , q * , r, s
Fronto-insula	1.2 (0.5)	0.9 (0.4)	1.5 (0.5)	1.7 (0.6)	1.7 (0.6)	1.7 (0.6)	1.6 (0.5)	2.1 (0.6)	2.3 (0.7)	2.0 (0.6)	c * , d, e * , f * , g, h * , i * , k * , l * , n * , q, s *
Anterior temporal	0.9 (0.5)	0.7 (0.5)	1.1 (0.4)	1.5 (0.5)	1.4 (0.5)	1.6 (0.5)	1.3 (0.4)	2.1 (0.9)	2.0 (0.9)	2.2 (0.9)	c * , d, e * , f * , g, h * , i * , k * , l * , n * , o * , r, s * , t *
Medial temporal	0.6 (0.6)	0.4 (0.4)	0.8 (0.7)	1.6 (0.9)	1.5 (0.9)	2.1 (0.9)	1.1 (0.7)	2.2 (0.9)	2.2 (1.0)	2.2 (1.0)	c * , d, e * , f * , g * , h * , i * , j, k * , l * , n, o * , p * , s * , t * , u
Posterior	0.9 (0.7)	0.7 (0.7)	1.1 (0.7)	1.6 (0.9)	1.8 (0.8)	1.3 (0.9)	1.2 (0.8)	1.3 (0.6)	1.4 (0.6)	1.3 (0.7)	c * , e, f * , h, i, m, u

Data are reported as mean (SD).

^a^ Pathological diagnosis was determined by biopsy in four patients with Alzheimer’s disease and three patients with FTLD patients; therefore, date of death was not available for these patients and they were not included in this analysis.

^b^ MMSE within 6 months of scan date was only available in 116/184 patients.

^c^ Alzheimer’s disease versus control.

^d^ DLB versus control.

^e^ FTLD versus control.

^f^ Early-onset Alzheimer’s disease versus younger controls.

^g^ Late-onset Alzheimer’s disease versus older controls.

^h^ FTLD-Tau versus younger controls.

^i^ FTLD-TDP43 versus younger controls.

^j^ Alzheimer’s disease versus DLB.

^k^ Alzheimer’s disease versus FTLD.

^l^ DLB versus FTLD.

^m^ Early-onset Alzheimer’s disease versus DLB.

^n^ Early-onset Alzheimer’s disease versus FTLD-Tau.

^o^ Early-onset Alzheimer’s disease versus FTLD-TDP43.

^p^ Late-onset Alzheimer’s disease versus DLB.

^q^ Late-onset Alzheimer’s disease versus FTLD-Tau.

^r^ Late-onset Alzheimer’s disease versus FTLD-TDP43.

^s^ DLB versus FTLD-Tau.

^t^ FTLD-Tau versus FTLD-TDP43.

^u^ Early-onset Alzheimer’s disease versus late-onset Alzheimer’s disease.

*lndicates significance at *P* < 0.001, otherwise *P* < 0.05; NA = not applicable.

In terms of the age-matched subgroups, the group of patients with late-onset Alzheimer’s disease was significantly older than the DLB group ( *P* < 0.05), with less time from scan until death ( *P* < 0.001). The group of patients with early-onset Alzheimer’s disease was significantly younger than the FTLD-Tau ( *P* < 0.001) and the FTLD-TDP43 ( *P* < 0.05) groups. They also scored significantly lower than the FTLD-Tau group on the MMSE ( *P* < 0.001).

### Expert diagnoses based on unstructured visual scan assessment

The mean sensitivity, specificity and balanced accuracy of expert diagnosis based on standard, unstructured assessments of the images are shown in Table 2 . Balanced accuracy was high (∼90%) for distinguishing Alzheimer’s disease and FTLD from controls, and ∼70% for DLB. For the more clinically relevant head-to-head disease comparisons balanced accuracy was on the order of 70–80% balanced accuracy for the multiple disease group comparisons was ∼60–70%, with specificities of 69–86%, but sensitivities were more variable, ranging from 34–67%.

**Table 2:** Accuracy of visual assessment for the primary pathology groups

	Unstructured visual assessment ^a^			Best single visual rating scale ^b^				SVC performance based on all scales ^c^		
**Classification task**	**Sensitivity**	**Specificity**	**Balanced accuracy**	**Best scale**	**Sensitivity**	**Specificity**	**Balanced accuracy**	**Sensitivity**	**Specificity**	**Balanced accuracy**
AD from controls	92% (7%)	86% (10%)	89% (6%)	MTA (1.5)	64% (56–72%)	89% (83–93%)	77% (69–83%)	94% (86–97%)	89% (80–94%)	92% (83–96%)
AD from DLB	84% (9%)	58% (17%)	71% (6%)	MTA (1.5)	64% (52–75%)	68% (56–78%)	66% (54–76%)	82% (66–91%)	64% (47–78%)	73% (56–85%)
AD from FTLD	81% (12%)	74% (11%)	77% (10%)	PA (2.5)	22% (15–30%)	98% (94–99%)	60% (50–69%)	88% (77–94%)	56% (42–68%)	72% (59–82%)
AD from DLB+FTLD	67% (13%)	69% (4%)	68% (8%)	PA (2.5)	22% (17–28%)	86% (81–90%)	54% (47–61%)	69% (57–78%)	68% (56–78%)	68% (57–78%)
DLB from controls	49% (20%)	92% (6%)	70% (9%)	OF (1.5)	57% (45–69%)	84% (73–90%)	70% (58–80%)	64% (46–79%)	92% (77–97%)	78% (60–89%)
DLB from AD	58% (17%)	84% (9%)	71% (6%)	OF (1.5)	57% (45–68%)	48% (36–59%)	52% (41–64%)	64% (47–78%)	82% (66–91%)	73% (56–85%)
DLB from FTLD	75% (26%)	89% (8%)	82% (12%)	PA (3.0)	7% (3–16%)	100% (95–100%)	54% (41–66%)	93% (78–98%)	89% (72–96%)	91% (75–97%)
DLB from AD+FTLD	34% (16%)	86% (6%)	60% (7%)	AC (1.0)	93% (85–97%)	11% (6–19%)	52% (41–63%)	93% (80–97%)	55% (39–70%)	74% (58–85%)
FTLD from controls	82% (5%)	99% (3%)	90% (2%)	MTA (1.5)	82% (73–88%)	89% (82–94%)	85% (77–91%)	89% (77–95%)	97% (89–99%)	93% (83–97%)
FTLD from AD	74% (11%)	81% (12%)	77% (10%)	OF (2.5)	55% (45–64%)	81% (73–87%)	68% (59–76%)	56% (42–68%)	88% (77–94%)	72% (59–82%)
FTLD from DLB	89% (8%)	75% (26%)	82% (12%)	MTA (2.0)	69% (56–79%)	82% (70–90%)	76% (63–85%)	89% (72–96%)	93% (78–98%)	91% (75–97%)
FTLD from AD+DLB	59% (9%)	80% (11%)	69% (6%)	AT (2.0)	64% (55–71%)	63% (54–71%)	63% (55–71%)	78% (66–86%)	68% (55–78%)	73% (60–82%)

^a^ Accuracy of expert diagnosis based on visual assessment of structural imaging ( *n* = 80, 20 per group). Experts were blinded to all clinical and pathological information except the person’s age. Data are presented as mean (SD) based on six dementia experts. Six raters, *n* = 80 scans.

^b^ Performance of visual rating scale that most accurately predicts pathology for each binary group comparison. The optimal cut-off points should be interpreted as: < cut-off = normal, ≥ cut-off = abnormal. Sensitivity and specificity values are selected based on the maximum balanced accuracy score. Four raters, *n* = 257 scans.

^c^ SVC performance based on mean left/right scores for each of the six visual rating scales. All values in parts (b) and (c) are based on mean scores from four raters in the 80-scan subset and two raters in the remaining images, and are presented with 95% confidence intervals in brackets. Four raters, *n* = 257 scans.

AD = Alzheimer’s disease.

### Time to perform visual rating

Mean time to perform and record all six visual rating scales based on three raters assessing the subset study population ( *n* = 80) was 2.9 ± 1.3 min. Individual rater means and standard deviations were 2.7 ± 1.1, 2.4 ± 1.0 and 3.6 ± 1.6 min.

### Inter-rater reliability of visual rating scores

Single measure and average measure ICC results for each scale are shown in Supplementary Table 1 . For the single measures ICC values, representing the reliability of each scale at the level of the individual rater, the MTA scale performed best overall, with very similar results achieved with two raters assessing all 257 scans, and four raters scoring 80 scans [ICC(2,1) ≥ 0.79]. The PA, OF and FI scales also demonstrated good reliability [ICC(2,1) ≥0.71] based on two raters assessing the total study population; reliability was slightly reduced when performed by four raters in the subset population [ICC(2,1) ≥ 0.58]. The reliability of the AC scale was lowest overall [ICC(2,1) range = 0.49–0.62]. As expected, the reliability based on mean rater scores was consistently greater for all scales [ICC(2,k) ≥ 0.73]. There were no material differences in reliability based on the larger or smaller population samples for any scale with the exception of the AT and AC scales, which were less reliable in the larger population sample.

### Correlation of grey matter volume with visual rating scores

Voxel-based morphometry analysis revealed a negative partial correlation of higher visual rating score with lower grey matter density for all visual rating scales. As shown in Fig. 1 , the pattern of regional atrophy correlated very closely with the specific brain region each scale was designed to assess. This regional specificity was highest for the MTA scale, although even the smaller frontal regions (OF and AC) showed significant correlation with their visual rating scales. Only the AT scale demonstrated a small region in the left superior parietal lobule/supramarginal gyrus where visual rating scores were positively correlated with grey matter atrophy (i.e. the reverse contrast; Supplementary Fig. 2 ). As higher AT scores are associated with FTLD pathologies, and in particular TDP43-C pathology associated with semantic dementia ( Whitwell *et al.* , 2010 ; Rohrer *et al.* , 2011 ), which are less likely to demonstrate atrophy in posterior brain regions, this result is pathologically plausible. As expected, analysis of each scale in separate models demonstrated a more diffuse pattern of atrophy, although, the most highly correlated regions were confined to, or included, the brain region targeted by each scale ( Supplementary Fig. 3 ).

### Mean visual rating scores per pathology group

The groups of patients with Alzheimer’s disease and FTLD both had significantly higher scores than the control group for all visual rating scales ( *P* < 0.05 AC in the Alzheimer’s disease comparison, *P* < 0.001 all other). The FTLD group also had significantly higher scores than the Alzheimer’s disease and DLB groups in all but the PA scale ( *P* < 0.001). Differences between the DLB and control groups did not reach significance for the AC and PA scales. The MTA scores for the DLB group were significantly lower than the Alzheimer’s disease group ( *P* < 0.05).

In terms of the age-matched subgroup comparisons, the young control group had significantly higher scores that the groups of patients with early-onset Alzheimer’s disease and FTLD ( *P* < 0.001, *P* < 0.05 PA in the FTLD comparisons). The FTLD-Tau group had significantly higher scores than the early-onset Alzheimer’s disease and DLB groups in all but the PA scale ( *P* < 0.05), and the late-onset Alzheimer’s disease group in the frontal scales only ( *P* < 0.001 AC, *P* < 0.05 OF, FI). The FTLD-TDP43 group had significantly higher scores than the early-onset Alzheimer’s disease and DLB groups based on the OF, AT and MTA scales ( *P* < 0.05), and the late-onset Alzheimer’s disease group based on the AC and AT scales ( *P* < 0.05). The late-onset Alzheimer’s disease group had significantly higher scores than the older controls in the OF, AT ( *P* < 0.05), and MTA scales ( *P* < 0.001). The late-onset Alzheimer’s disease group also had significantly higher scores than the DLB group in the MTA scale ( *P* < 0.05).

### Pathology classification accuracy for each visual rating scale

The results for the best performing scale for each group comparison are summarized in Table 2 . The MTA scale was most effective at accurately identifying Alzheimer’s disease pathology from the control group (AUC = 0.82) and the DLB group (AUC = 0.67). Higher PA scale scores (≥2.5) added some value in comparisons with the FTLD group, although sensitivity was low (22%). The OF scale was useful for distinguishing DLB from the control group (AUC = 0.74). All other scales were at chance for detecting DLB from the other disease groups. The MTA scale was the most effective at identifying FTLD pathology when compared with the control group (AUC = 0.92) and the DLB group (AUC = 0.81). Higher OF scale scores (≥2.5) were specific for FTLD pathology (81%) when compared with the Alzheimer’s disease group (AUC = 0.73).

Subgroup analysis is presented in Table 3 . Using age-matched controls improved accuracy in all comparisons except DLB versus older controls, which was slightly reduced (AUC = 0.74 to 0.70). In the early-onset Alzheimer’s disease versus younger control group, the FI scale was the single best scale for distinguishing between the groups, although the PA scale also performed well (cut-off = 1.5, AUC = 0.85). Otherwise, the PA scale was best for accurately identifying early-onset Alzheimer’s disease from the other groups, with the optimal cut-off varying by comparison. The MTA scale was the best single scale for identifying late-onset Alzheimer’s disease, performing well in the comparison with the DLB group (AUC =0.79), although at chance for the comparisons with the FTLD groups (FTLD-Tau: AUC = 0.50, FTLD-TDP43: AUC =0.45). Accuracy based on the highest left/right score rather than mean produced similar results, which are presented in Supplementary Table 2 .

**Table 3:** Accuracy of visual rating for the pathology subgroups

	Best single visual rating scale ^a^					SVC performance based on all scales ^b^			
Classification task	Best scale	Sensitivity	Specificity	Balanced accuracy	AUC	Sensitivity	Specificity	Balanced accuracy	AUC
EO-AD from younger controls	FI (1.5)	74% (63–83%)	94% (86–97%)	84% (74–90%)	0.89 (0.80–0.94)	86% (71–94%)	100% (91–100%)	93% (80–98%)	0.98 (0.87–1.0)
EO-AD from LO-AD	PA (1.5)	75% (63–84%)	61% (48–72%)	68% (56–78%)	0.68 (0.56–0.78)	67% (49–80%)	79% (61–89%)	73% (55–85%)	0.79 (0.61–0.89)
EO-AD from DLB	PA (2.0)	53% (41–65%)	75% (63–84%)	64% (52–75%)	0.68 (0.55–0.78)	86% (70–94%)	71% (53–84%)	79% (61–89%)	0.80 (0.63–0.90)
EO-AD from FTLD-Tau	PA (2.0)	53% (40–66%)	71% (58–81%)	62% (49–74%)	0.65 (0.52–0.76)	78% (59–89%)	50% (32–68%)	64% (45–79%)	0.56 (0.38–0.73)
EO-AD from FTLD-TDP43	PA (2.5)	26% (17–38%)	100% (95–100%)	63% (50–74%)	0.64 (0.52–0.75)	67% (49–80%)	71% (53–84%)	69% (51–82%)	0.73 (0.56–0.85)
EO-AD from LO-AD+DLB+FTLD	PA (1.5)	75% (68–81%)	38% (31–46%)	57% (49–64%)	0.60 (0.52–0.67)	56% (43–67%)	83% (73–90%)	69% (57–79%)	0.70 (0.58–0.79)
LO-AD from older controls	MTA (1.5)	82% (69–90%)	80% (67–88%)	81% (68–89%)	0.86 (0.74–0.93)	50% (32–68%)	100% (88–100%)	75% (55–87%)	0.78 (0.58–0.89)
LO-AD from EO-AD	MTA (2.0)	68% (55–78%)	64% (52–75%)	66% (54–76%)	0.69 (0.56–0.79)	79% (61–89%)	67% (49–80%)	73% (55–85%)	0.79 (0.61–0.89)
LO-AD from DLB	MTA (1.5)	82% (68–90%)	68% (53–80%)	75% (60–85%)	0.79 (0.64–0.88)	43% (25–64%)	93% (74–98%)	68% (46–83%)	0.66 (0.45–0.82)
LO-AD from FTLD-Tau	MTA (2.0)	68% (52–80%)	42% (28–57%)	55% (39–69%)	0.50 (0.35–0.65)	86% (64–95%)	58% (37–77%)	72% (49–87%)	0.71 (0.49–0.86)
LO-AD from FTLD-TDP43	MTA (0.5)	96% (87–99%)	4% (1–13%)	50% (36–64%)	0.45 (0.31–0.60)	86% (65–95%)	79% (57–90%)	82% (61–93%)	0.87 (0.66–0.95)
LO-AD from EOAD+DLB+FTLD	MTA (2.0)	68% (57–77%)	53% (42–64%)	60% (49–71%)	0.61 (0.50–0.71)	57% (41–72%)	84% (69–92%)	71% (54–83%)	0.71 (0.55–0.83)
DLB from (Older) Controls	OF (1.5)	57% (43–70%)	83% (70–90%)	70% (56–80%)	0.70 (0.56–0.81)	86% (67–94%)	40% (24–60%)	63% (43–79%)	0.53 (0.34–0.70)
DLB from EO-AD	OF (1.5)	57% (45–69%)	48% (36–60%)	53% (40–64%)	0.50 (0.38–0.63)	71% (53–84%)	86% (70–94%)	79% (61–89%)	0.80 (0.63–0.90)
DLB from LO-AD	AC (0.5)	100% (93–100%)	7% (3–18%)	54% (39–68%)	0.54 (0.39–0.68)	93% (74–98%)	43% (25–64%)	68% (46–83%)	0.66 (0.45–0.82)
DLB from FTLD-Tau	PA (3.0)	7% (3–19%)	100% (92–100%)	54% (38–68%)	0.45 (0.30–0.60)	86% (64–95%)	67% (44–83%)	76% (54–89%)	0.76 (0.53–0.89)
DLB from FTLD-TDP43	PA (2.5)	7% (3–18%)	100% (93–100%)	54% (39–68%)	0.44 (0.30–0.59)	71% (50–86%)	100% (86–100%)	86% (65–95%)	0.90 (0.70–0.97)
DLB from AD+FTLD	AC (1.0)	93% (85–97%)	10% (6–19%)	52% (41–63%)	0.43 (0.33–0.54)	86% (71–93%)	59% (43–73%)	72% (56–84%)	0.76 (0.60–0.86)
FTLD-Tau from younger controls	MTA (1.0)	100% (93–100%)	82% (68–90%)	91% (79–96%)	0.98 (0.89–1.0)	100% (85–100%)	100% (85–100%)	100% (85–100%)	1.0 (0.85–1.0)
FTLD-Tau from EO-AD	OF (2.0)	83% (72–91%)	59% (46–71%)	71% (58–81%)	0.76 (0.63–0.85)	50% (32–68%)	78% (59–89%)	64% (45–79%)	0.56 (0.38–0.73)
FTLD-Tau from LO-AD	AC (2.0)	58% (43–72%)	86% (72–93%)	72% (56–83%)	77% (61–87%)	58% (37–77%)	86% (64–95%)	72% (49–87%)	0.71 (0.49–0.86)
FTLD-Tau from DLB	MTA (1.5)	79% (64–89%)	68% (52–80%)	74% (58–84%)	0.80 (0.65–0.89)	67% (44–83%)	86% (64–95%)	76% (54–89%)	0.76 (0.53–0.89)
FTLD-Tau from FTLD-TDP43	FI (3.0)	33% (21–49%)	86% (72–93%)	60% (44–73%)	0.62 (0.46–0.75)	86% (64–95%)	17% (6–39%)	51% (31–71%)	0.40 (0.22–0.62)
FTLD-Tau from AD+DLB+FTLD-TDP43	FI (2.5)	50% (38–62%)	78% (67–86%)	64% (52–75%)	0.69 (0.57–0.79)	50% (34–66%)	81% (64–90%)	65% (48–79%)	0.62 (0.45–0.77)
FTLD-TDP43 from younger controls	FI (1.5)	93% (82–97%)	94% (84–98%)	93% (83–97%)	0.96 (0.87–0.99)	93% (75–98%)	100% (86–100%)	96% (80–99%)	0.98 (0.83–1.0)
FTLD-TDP43 from EO-AD	AT (2.0)	68% (55–78%)	73% (60–82%)	70% (58–80%)	0.76 (0.64–0.85)	71% (53–84%)	67% (49–80%)	69% (51–82%)	0.73 (0.56–0.85)
FTLD-TDP43 from LO-AD	AC (2.0)	54% (39–68%)	86% (72–93%)	70% (54–81%)	71% (56–82%)	79% (57–90%)	86% (65–95%)	82% (61–93%)	0.87 (0.66–0.95)
FTLD-TDP43 from DLB	AT (2.0)	68% (53–80%)	86% (72–93%)	77% (62–87%)	0.81 (0.67–0.90)	100% (86–100%)	71% (50–86%)	86% (65–95%)	0.90 (0.70–0.97)
FTLD-TDP43 from FTLD-Tau	MTA (2.0)	75% (59–86%)	42% (28–57%)	58% (43–72%)	0.55 (0.39–0.69)	17% (6–39%)	86% (64–95%)	51% (31–71%)	0.40 (0.22–0.62)
FTLD-TDP43 from AD+DLB+FTLD-Tau	AT (2.0)	68% (57–77%)	64% (52–73%)	66% (54–75%)	0.69 (0.58–0.78)	71% (55–83%)	70% (53–82%)	71% (54–83%)	0.72 (0.56–0.84)

^a^ Performance of visual rating scale that most accurately predicts pathology for each binary subgroup comparison. The optimal cut-off points should be interpreted as: < cut-off = normal, ≥ cut-off = abnormal. Sensitivity and specificity values are selected based on the maximum balanced accuracy score. Four raters, *n* = 254 scans*.

^b^ SVC performance based on mean left/right scores for each of the six visual rating scales. All values in parts (a) and (b) are presented with 95% confidence intervals in brackets. Four raters, *n* = 254 scans*.

*Three scans from patients with a primary pathology diagnosis of FTLD-FUS were excluded from the classification analysis due to insufficient sample size; AD = Alzheimer’s disease; EO = early onset; LO = late-onset.

### Support vector classification accuracy for pathology based on visual rating scores

**Figure 2:**
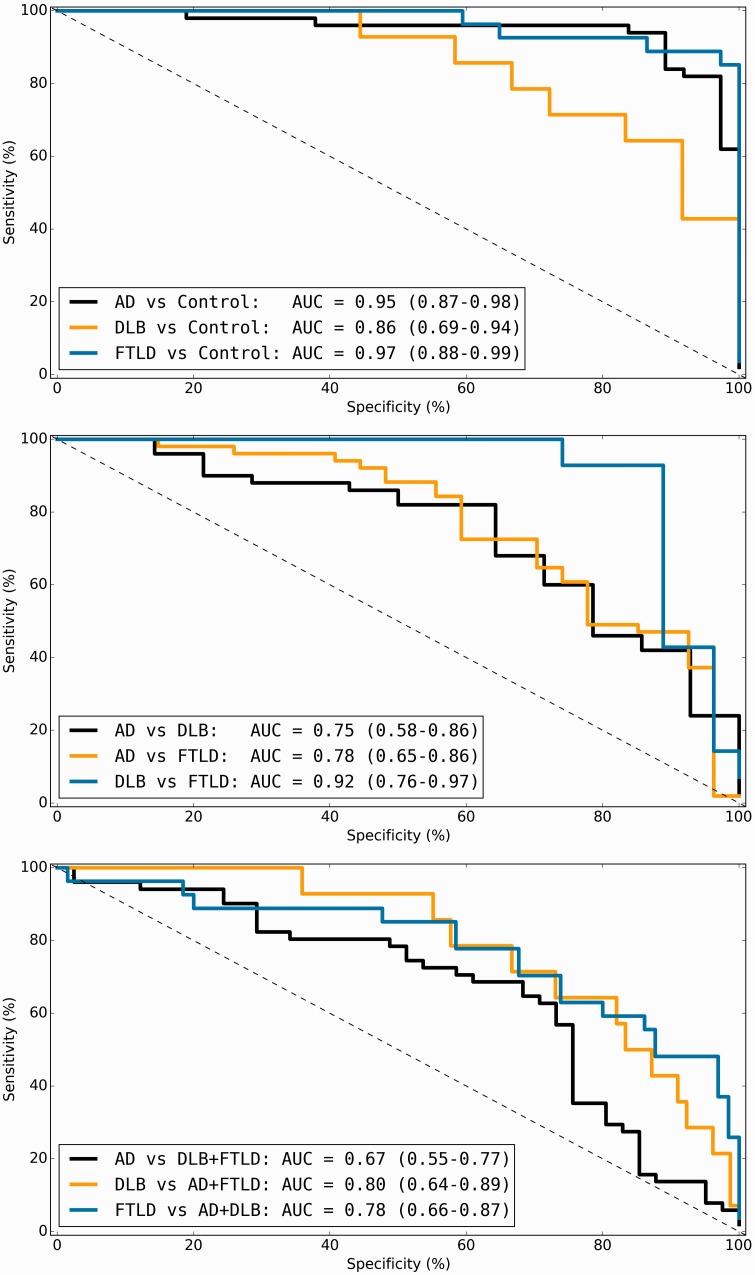
**Receiver operator characteristic curves of support vector machine performance.** Receiver operator characteristic plots of SVC performance for prediction of primary pathology groups. AUC values with 95% confidence intervals are displayed for each classifier. AD = Alzheimer’s disease.

The results for each group comparison using the mean right/left scores (i.e. six features) are summarized in Table 2 and illustrated in Fig. 2 . For all comparisons, the balanced accuracy ranged from 68–93%, and AUC from 0.67–0.97. SVC classification accuracy demonstrated a substantial improvement over the best single score in all cases, equivalent to or better than expert diagnosis. Based on the feature weighting applied by each SVC, the MTA, PA and AT scales contributed most to the separation of the Alzheimer’s disease group from controls, and the OF and AT scales contributed most to the separation of the DLB group from controls. With the exception of the PA scale, most scales contributed equally to the separation of the FTLD group from the controls. The PA (indicating Alzheimer’s disease), AT and OF scales (indicating FTLD) contributed most to the separation of the Alzheimer’s disease and FTLD groups. All scales except the PA scale contributed similarly to the separation of DLB and FTLD, weighted towards the FTLD group.

SVC accuracy based on the subgroup analysis is presented in Table 3 . In most cases the SVC improved the classification accuracy over the single scale, however, in some cases particularly comparisons involving the FTLD-Tau group the accuracy was reduced. Feature weighting for each SVC is displayed in Supplementary Table 3 . SVC performance based on individual left and right-sided hemisphere scores for each scale (12 features) are presented in Supplementary Table 2 , this improved the classification accuracy in 4 of the 10 SVCs that underperformed in comparison with the single scale (late-onset Alzheimer’s disease from DLB, DLB from (older) controls, FTLD-Tau from FTLD-TDP43, FTLD-TDP43 from FTLD-Tau).

## Discussion

This large, multi-centre study of pathologically proven dementias demonstrates that visual rating scales from routinely acquired structural MRIs are fairly reliable and highly correlated with cerebral atrophy in brain regions vulnerable to dementia pathology. When combined in an automated SVC, and in some cases when applied in isolation, they can be used to achieve diagnostic accuracy equivalent to, and in some cases better than, unstructured scan evaluation performed by expert raters. The rating scales in question are quick and easy to learn and can be applied, in total, in less than 3 min. Taken together, these results suggest that visual rating scales offer clinicians without expertise in neuroradiology a means of extracting diagnostically useful information in a time-efficient and inexpensive way that is ideally suited for integration into routine clinical practice.

The first aim of this study was to compare the inter-rater reliability of visual rating scales designed to assess cerebral atrophy in regions particularly vulnerable to the effects of dementia pathology. Although reliability is typically reported in the original concept study of each visual rating scale, and occasionally in follow-up studies, a lack of standardization in the use and reporting of statistical techniques employed to calculate this metric make it difficult to make direct comparisons ( Harper *et al.* , 2015 ). In this study, reliability was investigated using a dataset that is larger and more representative (through the inclusion of multiple dementia pathologies and ‘real life’ scans acquired on multiple scanners over many years) than is typically used for this purpose. The MTA scale was consistently highly reliable under all conditions. Of the adapted frontal scales, reliability was higher between the two raters assessing images from the total study population, than in the smaller sample rated by four. This difference is likely to reflect both the differences in sample sizes, and that the two raters (G.F. and L.H.) had more experience with these scales, suggesting that training may improve reliability. The reliability of the AC scale was lower overall, perhaps reflecting the sulcal variability in this rostral region, which can make it difficult to consistently identify the specified region of interest. The PA scale, requiring the integration of visual information in three planes in four brain regions (parietal lobe, posterior cingulate sulcus, parieto-occipital sulcus and precuneus), is undoubtedly the most difficult to apply. Despite this, the two raters assessing the total study population achieved a relatively high degree of reliability, although there was more variability among the four raters based on the smaller dataset. The consistently high reliability of the average measures ICC (based on mean scale scores averaged over raters) perhaps suggests that where possible, the use of mean scores from two or more raters may be preferable when practicable.

Using voxel-based morphometry, each of the scales was remarkably well correlated with the anatomical regions of interest they were designed to assess, illustrating their regional specificity. This was particularly true for the MTA scale, which was highly associated with hippocampal volume loss, but even the more complex PA scale was well correlated with the posterior pattern of atrophy it was designed to detect. Focal atrophy in the small frontal regions assessed by the OF scale, and to a lesser extent the AC scale (right side only), was also significantly correlated with their associated visual rating scores. While previous studies have investigated the relationship between rating scales and brain volumes in the region of interest ( Davies *et al.* , 2009 ; Shen *et al.* , 2011 ; Moller *et al.* , 2014 ), these results using an unbiased technique provide independent validation that each of the scales is indeed performing as predicted. The concordance can also be considered as evidence that voxel-based morphometry (as implemented in SPM12b) is performing well in this challenging heterogeneous dataset.

While several studies have estimated the classification accuracy of rating scales in the diagnosis of various dementias ( Scheltens *et al.* , 1992 ; Galton *et al.* , 2001 ; Barkhof *et al.* , 2007 ; Kipps *et al.* , 2007 ; Burton *et al.* , 2009 ; Davies *et al.* , 2009 ; Duara *et al.* , 2010 ; Hornberger *et al.* , 2010 ; Koedam *et al.* , 2011 ; Lehmann *et al.* , 2012 ; Ambikairajah *et al.* , 2014 ; Moller *et al.* , 2014 ; Harper *et al.* , 2015 ), very few have used histopathological diagnosis as the gold standard ( Likeman *et al.* , 2005 ; Barkhof *et al.* , 2007 ; Burton *et al.* , 2009 ; Lehmann *et al.* , 2012 ), and to our knowledge no study has performed this analysis in such a large, clinically realistic cohort comprising as wide a range of diverse pathologies and made comparisons with expert scan assessment.

There were significant differences between group mean scores in both the primary group analysis (Alzheimer’s disease, DLB, FTLD and control) and the subgroup analysis (early and late-onset Alzheimer’s disease, DLB, FTLD-Tau, FTLD-TDP43, younger and older controls) for all visual rating scales. Specifically, the early-onset Alzheimer’s disease and FTLD group scores were significantly different from the younger control group for all scales, reflecting the vulnerability of these regions to dementia pathology. However, higher scores in the older control group compared to the younger control group, reaching significance in the FI and AT scale ( *P* < 0.05), underline the need to account for age in the visual assessment of structural brain imaging. Recent work by Ferreira *et al.* (2015 ), and previous reports from Duara *et al.* (2013 ) and Barkhof et al. (2007) to define age-specific cut-offs for the MTA and PA scales may help to address this issue for these scales, but to our knowledge similar values have not yet been defined for the frontal scales. Incorporating the visual rating scale scores into an automated classifier, however, allows age to be accounted for more easily ( Coupé *et al.* , 2012 ; Koikkalainen *et al.* , 2012 ) and removes the requirement for such cut-offs.

As previous studies have shown, the MTA and PA scales were the most useful for predicting Alzheimer’s disease pathology ( Scheltens *et al.* , 1992 ; Burton *et al.* , 2009 ; Koedam *et al.* , 2011 ; Lehmann *et al.* , 2012 ). The MTA scale was specifically useful for late-onset Alzheimer’s disease, while the PA scale was more useful for younger early-onset Alzheimer’s disease, which typically results in a higher proportion of non-amnestic, atypical presentations ( Rossor *et al.* , 2010 ). Classification of the early-onset Alzheimer’s disease group in comparison with the FTLD groups, based on the PA scale, was similar to previous reports in the literature [AUC = 0.63 (Tau), AUC = 0.67 (TDP-43) versus AUC = 0.66 (mixed FTLD) ( Lehmann *et al.* , 2012 )]. However, in the comparison with the FTLD-TDP43 group, a higher cut-off score (≥2.5 versus ≥2; Koedam *et al.* , 2011 ; Lehmann *et al.* , 2012 ) was required to provide optimal separation, compromising sensitivity (22%) at the expense of higher specificity. Using the MTA scale, separation of the group of patients with late-onset Alzheimer’s disease from the DLB group was better than the equivalent comparison with the early-onset group using the PA scale (AUC = 0.76 versus 0.66). This result is in keeping with evidence from previous studies suggesting that DLB pathology is relatively sparing of the hippocampi in comparison to Alzheimer’s disease. There was no single optimal scale for distinguishing between the groups of patients with FTLD; however, the frontal scales (OF, AC and FI) were best for distinguishing the FTLD-Tau group, while temporal scales (AT and MTA) were best for distinguishing the FTLD-TDP43 group. Using higher OF scale scores to distinguish between Alzheimer’s disease and FTLD (≥2.5) has previously been described by Hornberger *et al.* (2010 ) in a much smaller cohort (AUC = 0.7 versus 0.75 in this study), however, we also found this scale to be useful in distinguishing DLB from healthy older controls (AUC = 0.74). To our knowledge, this is a novel use of this scale not previously explored in other studies and echoes findings in earlier work ( Förstl *et al.* , 1993 ) of marked and disproportionate frontal atrophy on CT images from autopsy confirmed DLB. While in some cases a single scale produces good classification accuracy for dementia pathology, in most cases, this is greatly improved by combining all scores in an automated classifier, producing diagnostic accuracy equivalent to, and in some cases better than, unstructured scan evaluation performed by dementia experts. Furthermore, accuracy of classification based on visual rating is also consistent with the reported accuracies from a previous study using grey matter volume to distinguish between cases of pathologically confirmed Alzheimer’s disease and FTLD ( Whitwell *et al.* , 2011 ). Given the ease and accuracy of applying these ratings, this approach provides a potentially valuable way for non-experts to extract valuable diagnostic information from routine scans.

While there is considerable interest in using molecular biomarker techniques to aid in the differential diagnosis, particularly of Alzheimer’s disease ( Ahmed *et al.* , 2014 ), it is notable that the classification accuracy we report is comparable to the accuracy of the CSF amyloid-β _1-42_ level as recently reported in a large sample (balanced accuracy: Alzheimer’s disease from DLB = 64%, Alzheimer’s disease from FTLD = 81%; Ewers *et al.* , 2015 ). Although these tests identify different aspects of the disease process, and noting that we used only the primary post-mortem diagnosis excluding co-pathology, each test’s contribution to an accurate differential diagnosis is similar. Continued optimization of the classifier through the inclusion of more data is likely to improve performance beyond what can be achieved with simple dichotomization of an individual scale. While the required level of data to achieve such classifiers is unlikely to be available within any single centre, pooling imaging and pathology data between centres and making them accessible online to predict pathology from rating scores could provide a communal resource that is useful for both research purposes and as a diagnostic aid in clinic.

This study has a number of strengths including the large overall sample size, use of multiple scales, post-mortem confirmation of diagnosis, ‘real life’ acquisition of scans, and comparisons based on blind assessment, i.e. without the benefit of clinical information, which in practice is likely to improve diagnostic performance. Limitations include the imbalance in the pathology groups, in particularly the relatively low number of DLB cases included, and the disproportionate representation of young-onset Alzheimer’s disease cases compared to the average clinical population. To obtain sample sizes sufficient for these analyses, we treated FTLD as a single diagnostic group, and then subdivided these cases by primary molecular pathology, however, further stratification and more fine-grained analysis was not possible. Control subjects were not pathologically confirmed, therefore, we cannot rule out presymptomatic pathology in this group, which would result in an underestimation of specificity. However, this does not affect the more clinically relevant between-pathology group comparisons. Whilst the sample size is large in the context of pathologically confirmed dementias, larger numbers in all groups would improve statistical certainty, particularly in the SVC experiments where it is necessary to split the data into training and testing sets. Greater power could also allow for more fine-grained analysis of subtypes of the canonical dementias, and for an investigation of the role of mixed or multiple pathologies. In terms of expert scan assessment, classification performance was based on a subset of the total study population and assumed to represent assessment of the entire dataset. Visual assessment was also performed in native space to better reflect clinical practice; however, reorientation to standard space would allow for greater anatomical consistency between scans and may potentially improve inter-rater reliability and diagnostic accuracy. Finally, while the raters participating in this study have considerable experience in the application of visual rating scales, it will be of interest to see if similar results can be obtained in less experienced, or indeed novice raters, and to assess the effects of training in these individuals.

In summary, this study demonstrates the utility of visual rating scales to provide diagnostically useful information, which when considered in the context of a detailed clinical examination may help to improve the accuracy of clinical diagnosis for the degenerative dementias. Visual rating offers a simple and reliable framework to capitalize on the structural imaging already acquired in most patients at no extra cost. Until more advanced image analysis techniques are adapted for use in clinical practice, the incorporation of visual rating scales (certainly when combined with an automated classifier) offers a quick, simple, reliable means of extracting valuable diagnostic information from structural brain imaging.

## Funding

The Dementia Research Centre is an Alzheimer's Research UK coordinating centre. The authors acknowledge the support of Alzheimer's Research UK [grant number ART-NCG2010B-2], the Medical Research Council [grant number MR/J014257/2], the NIHR Queen Square Dementia Biomedical Research Unit, UCL/H Biomedical Research Centre, the Leonard Wolfson Experimental Neurology Centre, the NIHR Newcastle Biomedical Research Unit in Lewy body dementia, and the NIHR Cambridge Biomedical Research Unit in Dementia. L.H. is supported by funding from Alzheimer's Research UK and a UCL Impact Studentship. G.F. is supported by a European Neurological Society Fellowship. N.C.F. and J.O.B. hold NIHR Senior Investigator Awards. G.R. is supported by the Medical Research Council. J.M.S acknowledges the support of the NIHR Queen Square Dementia BRU, the NIHR UCL/H Biomedical Research Centre, Wolfson Foundation, EPSRC (EP/J020990/1), MRC (CSUB19166), ARUK (ARUK-Network 2012-6-ICE; ARUK-PG2014-1946), and European Commission (H2020-PHC-2014-2015-666992). JR is an MRC Clinician Scientist and also receives funding from the NIHR Rare Diseases Translational Research Collaboration.

## Supplementary material

Supplementary material is available at *Brain* online.

## Supplementary Material

Supplementary Data

## Abbreviations

AC: anterior cingulate
AUC: area under the receiver-operator characteristic curve
DLB: dementia with Lewy bodies
FI: fronto-insula
FTLD: frontotemporal lobar degeneration
ICC: intraclass correlation coefficient
ICC(2,1): two-way single measures ICC
ICC(2,k): two-way average measures ICC based on k raters
MTA: medial temporal lobe atrophy
OF: orbito-frontal
PA: posterior atrophy
SVC: support vector classifier
