# Supplementary material for: MRI visual rating scales in the diagnosis of dementia: evaluation in 184 post-mortem confirmed cases
Source: Brain. 2016 Mar 1;139(4):1211–25. doi: 10.1093/brain/aww005 (PMC4806219; doi:10.1093/brain/aww005)
Supplement: Supplementary Data [file aww005_supplementary_data.zip › brain-2015-01186-File016.pdf]

Orbito-Frontal Rating Protocol

Slice Selection

a) Corpus callosum not yet visible (pre-rating slice)
b) Corpus callosum just visible (rate olfactory sulcus and cingulate sulcus on this slice)
c) Post-rating slice

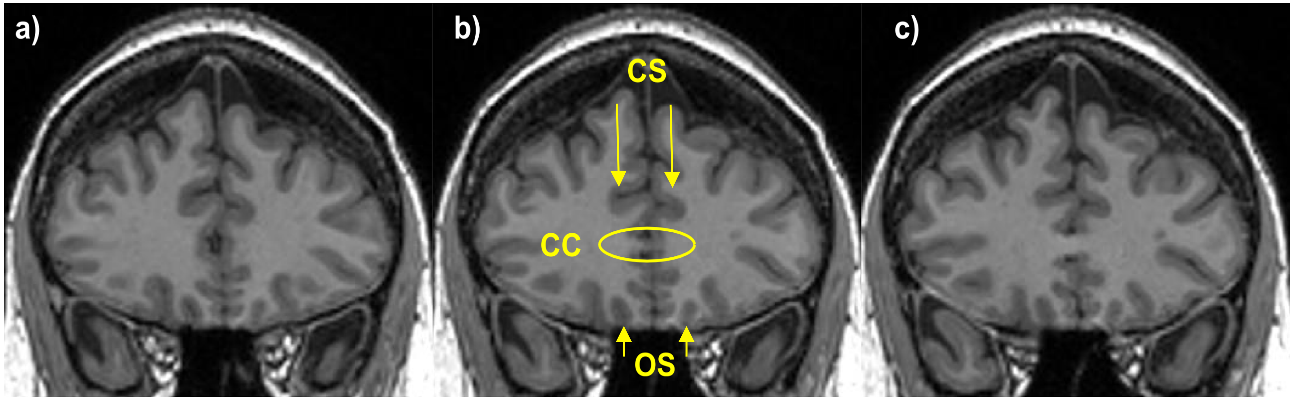

CC - corpus callosum, OS - olfactory sulcus, CS - cingulate sulcus

Rating Guide

0: Closed sulcus
1: Small sulcal slit, just revealing CSF
2: Opening of the sulcus, CSF clearly visible
3: Severe widening of the sulcus

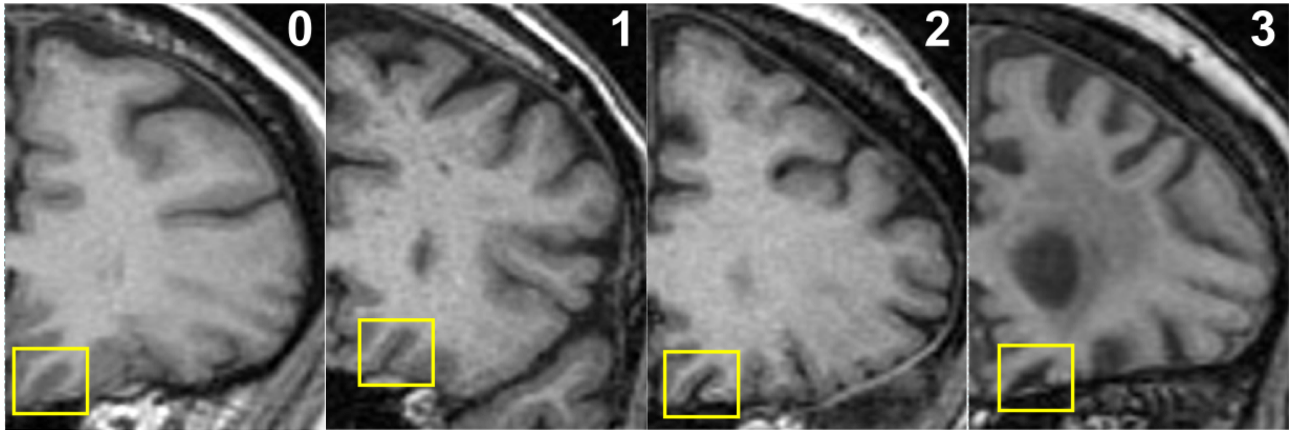

Rostral Anterior Cingulate Rating Protocol

Slice Selection

a) Corpus callosum not yet visible (pre-rating slice)
b) Corpus callosum just visible (rate olfactory sulcus and cingulate sulcus on this slice)
c) Post-rating slice

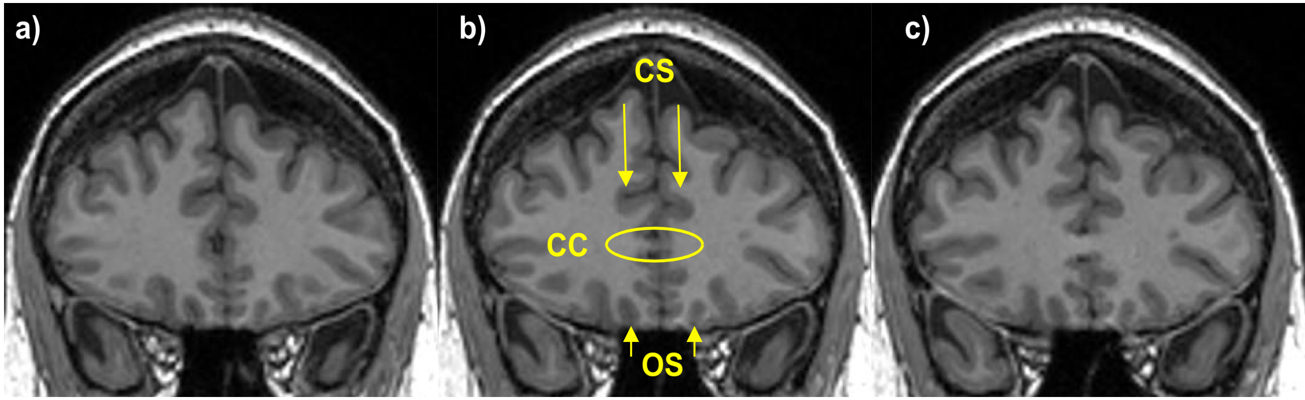

CC - corpus callosum, OS - olfactory sulcus, CS - cingulate sulcus

Rating Guide

0: Closed sulcus
1: Sulcal opening (CSF visible), although narrower towards the peak
2: Sulcal widening along the length of the sulcus
3: Severe widening of the sulcus

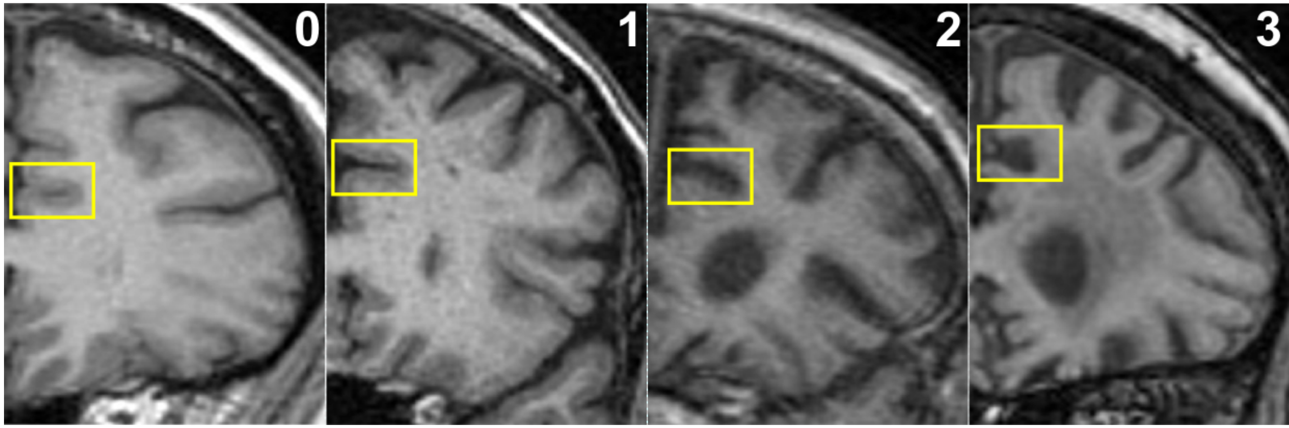

Anterior Temporal Rating Protocol

Slice Selection

a) Connection between the frontal and temporal lobes is still visible (pre-rating slice)
b) No visible connection between the frontal and temporal lobes (rate this slice)
c) Post-rating slice

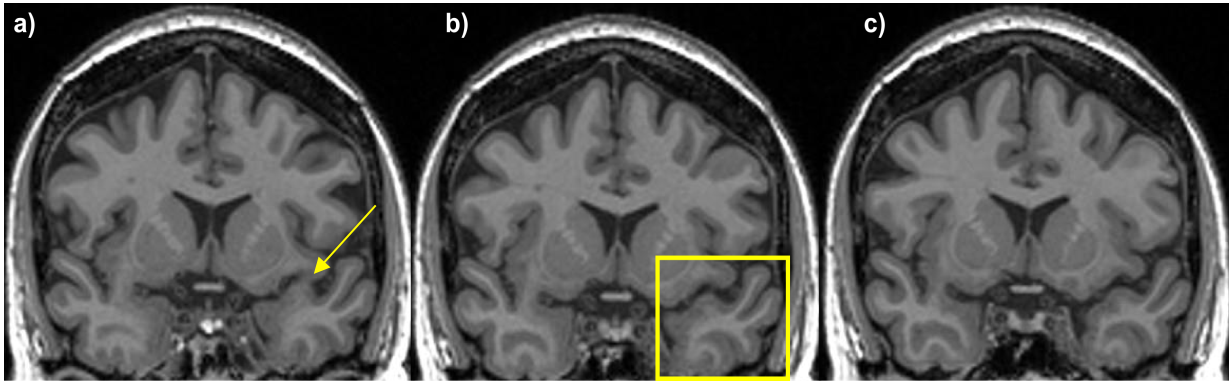

Rating Guide

0: Normal appearances
1: Slight prominence of anterior temporal sulci
2: Temporal sulci definitely widened
3: Gyri severely atrophic and ribbon-like. WM and GM cannot be distinguished (normal temporal lobe at this level is less substantial than the frontal lobe, ribbon-like gyri of stage 3 temporal lobe are similar to stage 4 frontal gyri)
4: Temporal pole has a simple linear profile or is not seen at all

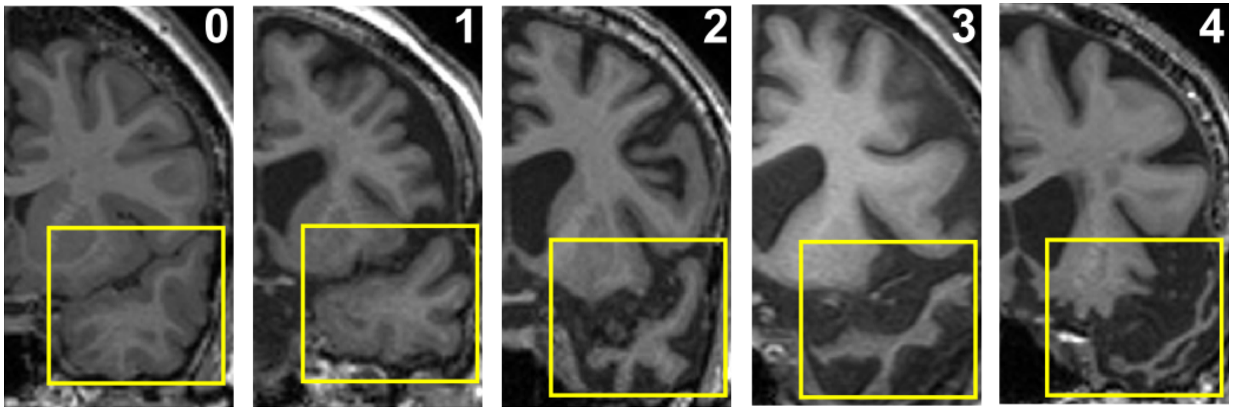

Fronto-Insula Rating Protocol

Slice Selection

a) Anterior commissure (AC) not yet visible (pre-rating slice)
b) Anterior commisure just visible (rate this slice and the 2 posterior)

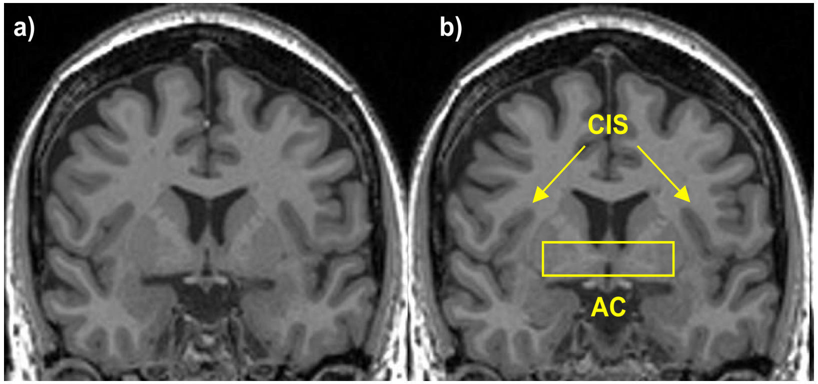

CIS: Circular insular sulcus, AC: Anterior commissure

Rating Guide

(Average the score over the 3 slices)

0: Closed sulcus
1: Sulcal opening, CSF clearly visible
2: Sulcal widening and the emergence of an arrow head shape pointing towards the midline
3: Severe widening along the length of the sulcus

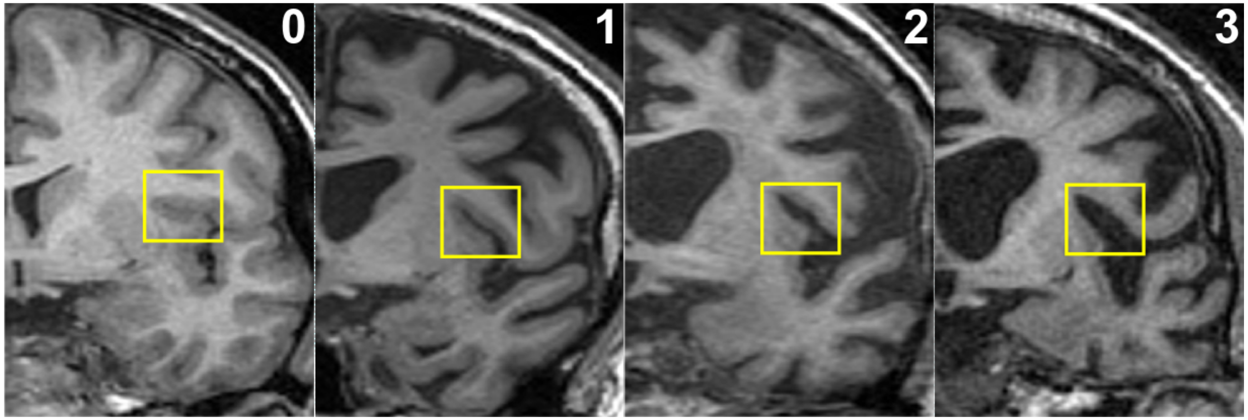

Medial Temporal Rating Protocol

Slice Selection

- In the middle of the hippocampal body, in front of the pons or halfway through the pons depending on the angle of the scan
- Scroll though the hippocampus to get an impression of the atrophy throughout
- Don't rate too close to the amygdala. If the hippocampus curls up, the slice is too close to the hippocampal head.
- At the origin of the fornix, the slice is too close to the tail.
- A score of 0 can still be given if there is some opening of the choroid fissure on a few slices through the hippocampal body if the remainder are closed.
- A score of 1 is given if the choroid fissure is opened over the entire length of the hippocampal body.

Rating Guide

(References images from http://www.radiologyassistant.nl/en/p43dbf6d16f98d/dementia-role-of-mri.html)

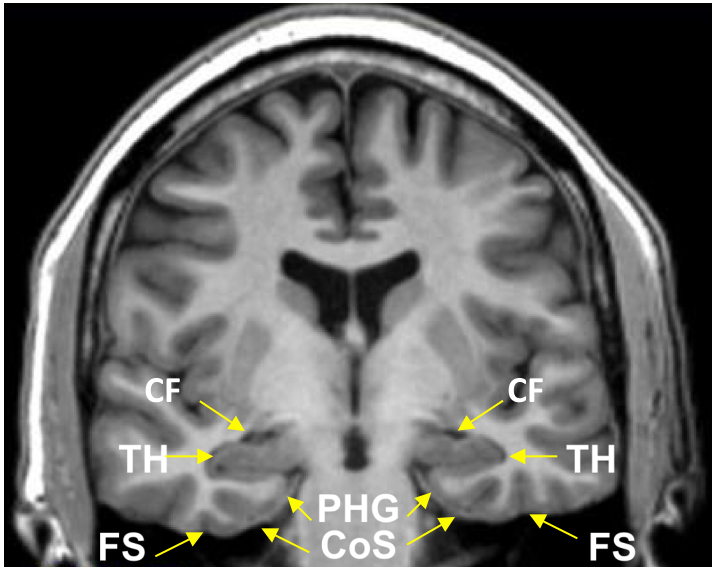

Also take these structures into account:
CF – choroid fissure
TH - temporal horn
PHG - parahippocampal gyrus
CoS - collateral sulcus
FS - fusiform sulcus

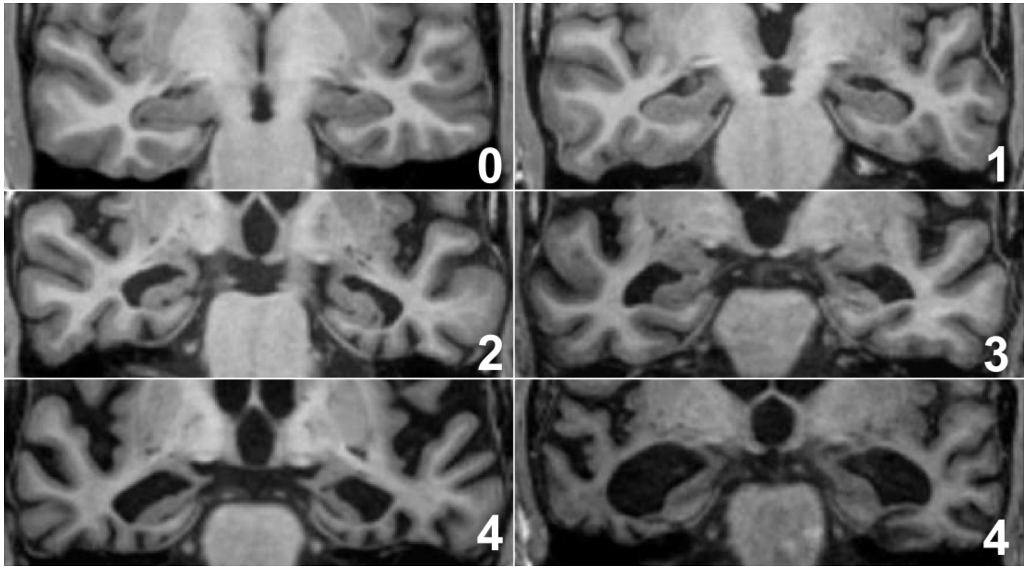

0: Normal,
1: Widened choroid fissure
2: Increased widening of the choroid fissure, widening of the temporal horn, opening of other sulci (i.e. collateral/fusiform sulcus)
3: Pronounced volume loss of the hippocampus,
4: End stage atrophy

Posterior Atrophy Rating Protocol

0

1

2

3

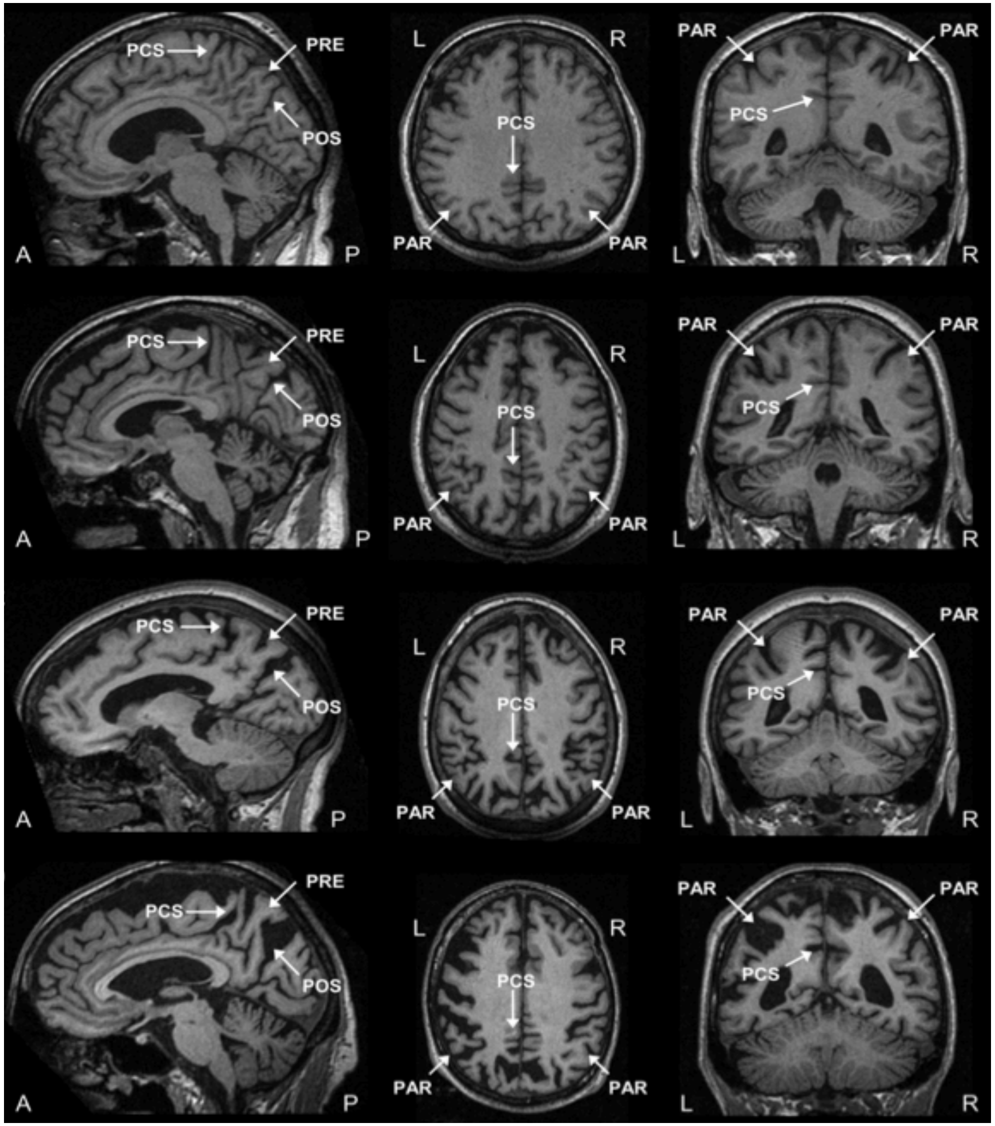

Rating Guide

0: Closed sulci of parietal lobes and cuneus
1: Mild widening of posterior cingulate and parieto-occipital sulci
2: Substantial widening of the sulci
3: Extreme widening of the posterior cingulate and parieto-occipital sulci

Slice Selection

No slice selection – just scroll through
PAR - parietal lobe
PCS - posterior cingulate sulcus
POS - parieto-occipital sulcus
PRE -precuneus

Image from Lehmann et al,
Neurobiol Aging. 2012 Mar;33(3):627.e1-627.e12.
