# Supplementary material for: MRI visual rating scales in the diagnosis of dementia: evaluation in 184 post-mortem confirmed cases
Source: Brain. 2016 Mar 1;139(4):1211–25. doi: 10.1093/brain/aww005 (PMC4806219; doi:10.1093/brain/aww005)
Supplement: Supplementary Data [file aww005_supplementary_data.zip › brain-2015-01186-File018.pdf]

Supplementary Table 1 Inter-rater reliability of visual rating scales. Intraclass correlation coefficient (ICC) measures with confidence intervals. Single-measures ICC provide an estimate of the reliability of the scale when applied by a single rater. The average-measures ICC is an estimate of the reliability of the scale based on an average score derived from k raters.

Supplementary Table 2 Accuracy of visual rating for the pathology subgroups based on highest left/right score. A. Performance of visual rating scale that most accurately predicts pathology for each binary subgroup comparison. The optimal cut-off points should be interpreted as: < cut-off = normal, ≥ cut-off = abnormal. Sensitivity and specificity values are selected based on the maximum balanced accuracy score. **B.** Support vector classifier (SVC) performance based on mean left/right scores for each of the six visual rating scales. All values in parts A and B are presented with 95% confidence intervals in brackets. AD = Alzheimer's disease, DLB = dementia with Lewy bodies, FTLD = frontotemporal lobar degeneration.

(a) Three scans from patients with a primary pathology diagnosis of FTLD-FUS were excluded from the classification analysis due to insufficient sample size.

Supplementary Table 3 Feature weighting for each binary comparison. Features are listed in order from greatest influence over group separation (w1) to least (w6). Negative values indicate influence on the opposite side of the separating hyperplane. AD = Alzheimer's disease, DLB = dementia with Lewy bodies, FTLD = frontotemporal lobar degeneration.

Supplementary Table 4 Distribution of pathology group per scanning site and magnetic field strength. AD = Alzheimer's disease, DLB = dementia with Lewy bodies, FTLD = frontotemporal lobar degeneration.

Supplementary Figure 1 Visual rating analysis streams. Flow chart indicating the visual rating analysis performed and the subsequent analysis streams. AD = Alzheimer's disease, EOAD = Early-onset AD, DLB = dementia with Lewy bodies, FTLD = frontotemporal lobar degeneration.

Supplementary Figure 2 Voxel-based morphometry-anterior temporal reverse contrast. VBM analysis of the anterior temporal scale demonstrated a small region in the left superior parietal lobule/supramarginal gyrus where visual rating scores were positively correlated with grey matter atrophy.

Supplementary Figure 3 Voxel-based morphometry-simple correlations. Six simple correlations of each scale with grey matter volume, e.g. $y = \beta_{AC}X_{AC} + \beta_{Age}X_{Age} + \beta_{Gender}X_{Gender} + \beta_{TIV}X_{TIV} + \beta_{1T}X_{1T} + \beta_{3T}X_{3T} + \beta_{London}X_{London} + \beta_{Amsterdam}X_{Amsterdam} + \mu + e$. In all images statistical significance of correlations was corrected for multiple comparisons (family wise error rate $P < 0.05$). The corresponding visual rating scale reference images are displayed adjacent to each statistical parametric map. R indicates the right hemisphere. TIV = total intracranial volume.

Supplementary Figure 4 Distribution of visual rating scores. Distribution of visual rating scores for each scale per primary pathology group. AD = Alzheimer's disease, DLB = dementia with Lewy bodies, FTLD = frontotemporal lobar degeneration.

Supplementary Figure 5 Differences in grey matter volume between pathology subgroups. Voxel-based morphometry images demonstrating differences in grey matter volume between pathology subgroups ($Y = \beta_{EOAD}X_{EOAD} + \beta_{LOAD}X_{LOAD} + \beta_{DLB}X_{DLB} + \beta_{FTLD\text{-}Tau}X_{FTLD\text{-}TDP43} + \beta_{YoungerControls}X_{YoungerControls} + \beta_{OlderControls}X_{OlderControls} + \beta_{Age}X_{Age} + \beta_{Gender}X_{Gender} + \beta_{TIV}X_{TIV} + \beta_{1T}X_{1T} + \beta_{3T}X_{3T} + \beta_{London}X_{London} + \beta_{Amsterdam}X_{Amsterdam} + \mu + e$). The slice demonstrating the global maximum difference is presented. Statistical significance is indicated for each row of images. FWE = family-wise error rate, UNC. = uncorrected, AD = Alzheimer's disease, EO = early-onset, LO = late-onset, DLB = dementia with Lewy bodies, FTLD = frontotemporal lobar degeneration.

Supplementary imaging protocol: The reference materials provided to each rater during image assessment.
