# Supplementary material for: MRI visual rating scales in the diagnosis of dementia: evaluation in 184 post-mortem confirmed cases
Source: Brain. 2016 Mar 1;139(4):1211–25. doi: 10.1093/brain/aww005 (PMC4806219; doi:10.1093/brain/aww005)
Supplement: Supplementary Data [file aww005_supplementary_data.zip › brain-2015-01186-File007.pdf]

Scale	Single-Measures ICC		Average-Measures ICC	
	**4 raters, *n* = 80**	**2 raters, *n* = 257**	**4 raters, *n* = 80**	**2 raters, *n* = 257**
Left MTA	0.82 (0.76 - 0.88)	0.84 (0.79 - 0.87)	0.95 (0.93 - 0.97)	0.91 (0.89 - 0.93)
Right MTA	0.79 (0.71 - 0.85)	0.83 (0.79 - 0.86)	0.94 (0.91 - 0.96)	0.91 (0.88 - 0.93)
Left PA	0.59 (0.48 - 0.70)	0.71 (0.65 - 0.77)	0.85 (0.79 - 0.90)	0.83 (0.79 - 0.87)
Right PA	0.65 (0.55 - 0.75)	0.72 (0.66 - 0.78)	0.88 (0.83 - 0.92)	0.84 (0.79 - 0.87)
Left AT	0.69 (0.57 - 0.78)	0.62 (0.53 - 0.70)	0.90 (0.84 - 0.94)	0.77 (0.70 - 0.82)
Right AT	0.62 (0.48 - 0.73)	0.57 (0.48 - 0.65)	0.87 (0.79 - 0.92)	0.73 (0.65 - 0.79)
Left OF	0.58 (0.46 - 0.70)	0.72 (0.65 - 0.78)	0.85 (0.77 - 0.90)	0.84 (0.79 - 0.88)
Right OF	0.61 (0.49 - 0.71)	0.74 (0.68 - 0.79)	0.86 (0.79 - 0.91)	0.85 (0.81 - 0.88)
Left AC	0.56 (0.45 - 0.67)	0.61 (0.51 - 0.69)	0.84 (0.76 - 0.89)	0.76 (0.68 - 0.82)
Right AC	0.49 (0.37 - 0.61)	0.62 (0.53 - 0.69)	0.80 (0.71 - 0.86)	0.76 (0.69 - 0.82)
Left FI	0.61 (0.50 - 0.72)	0.73 (0.67 - 0.79)	0.86 (0.80 - 0.91)	0.85 (0.80 - 0.88)
Right FI	0.61 (0.50 - 0.71)	0.72 (0.65 - 0.77)	0.86 (0.80 - 0.91)	0.84 (0.79 - 0.87)
