# Supplementary material for: MRI visual rating scales in the diagnosis of dementia: evaluation in 184 post-mortem confirmed cases
Source: Brain. 2016 Mar 1;139(4):1211–25. doi: 10.1093/brain/aww005 (PMC4806219; doi:10.1093/brain/aww005)
Supplement: Supplementary Data [file aww005_supplementary_data.zip › brain-2015-01186-File008.pdf]

Classification Task	Best Scale	A. Best Single Visual Rating Scale (4 raters, n = 254 scans[a]) Sensitivity	Specificity	Balanced Accuracy	AUC	B. SVC Performance Based on All Scales (4 raters, n = 254 scans[a]) Sensitivity	Specificity	Balanced Accuracy	AUC
EOAD from (Younger) Controls	FI (1.5)	74% (63-83%)	94% (86-97%)	84% (74-90%)	0.86 (0.76-0.92)	92% (78-97%)	100% (91-100%)	96% (84-99%)	0.98 (0.87-1.0)
EOAD from LOAD	PA (1.5)	78% (66-86%)	61% (48-72%)	69% (57-79%)	0.68 (0.56-0.78)	86% (69-94%)	57% (40-73%)	72% (54-84%)	0.78 (0.60-0.88)
EOAD from DLB	PA (1.5)	78% (66-86%)	43% (31-55%)	60% (48-72%)	0.66 (0.53-0.76)	89% (73-96%)	71% (53-84%)	80% (63-90%)	0.81 (0.64-0.91)
EOAD from FTLD-Tau	PA (1.5)	78% (66-87%)	42% (30-55%)	60% (47-72%)	0.63 (0.50-0.75)	86% (69-94%)	42% (25-60%)	64% (45-79%)	0.58 (0.40-0.75)
EOAD from FTLD-TDP43	PA (2.5)	26% (17-38%)	100% (95-100%)	63% (50-74%)	0.67 (0.55-0.77)	47% (31-64%)	93% (79-98%)	70% (52-83%)	0.72 (0.55-0.85)
EOAD from LOAD+DLB+FTLD	PA (1.5)	78% (71-84%)	37% (30-45%)	58% (50-65%)	0.60 (0.52-0.67)	69% (57-79%)	65% (53-75%)	67% (55-77%)	0.69 (0.57-0.78)
LOAD from (Older) Controls	MTA (1.5)	82% (69-90%)	80% (67-88%)	81% (68-89%)	0.86 (0.74-0.93)	57% (37-74%)	100% (88-100%)	79% (59-90%)	0.80 (0.61-0.91)
LOAD from EOAD	MTA (1.5)	82% (71-89%)	42% (31-55%)	62% (50-73%)	0.67 (0.55-0.77)	57% (40-73%)	86% (70-94%)	72% (54-84%)	0.78 (0.60-0.88)
LOAD from DLB	MTA (1.5)	82% (68-90%)	68% (53-80%)	75% (60-85%)	0.76 (0.61-0.86)	50% (30-70%)	93% (74-98%)	71% (50-86%)	0.77 (0.55-0.89)

LOAD<br>from FTLD-Tau	MTA<br>(1.5)	82%<br>(67-91%)	21%<br>(11-36%)	51%<br>(36-66%)	0.49<br>(0.34-0.64)	79%<br>(56-91%)	67%<br>(44-83%)	73%<br>(50-87%)	0.73<br>(0.50-0.87)
LOAD<br>from FTLD-TDP43	MTA<br>(0.5)	96%<br>(87-99%)	4%<br>(1-13%)	50%<br>(36-64%)	0.46<br>(0.32-0.60)	64%<br>(43-81%)	100%<br>(86-100%)	82%<br>(61-93%)	0.81<br>(0.59-0.92)
LOAD<br>from EOAD+DLB+FTLD	MTA<br>(1.5)	82%<br>(72-89%)	36%<br>(26-47%)	59%<br>(48-69%)	0.60<br>(0.49-0.70)	50%<br>(34-66%)	91%<br>(77-96%)	70%<br>(54-82%)	0.70<br>(0.54-0.82)
DLB<br>from (Older) Controls	OF<br>(1.5)	61%<br>(47-73%)	83%<br>(70-90%)	72%<br>(58-82%)	0.75<br>(0.61-0.84)	57%<br>(38-74%)	90%<br>(73-97%)	74%<br>(54-86%)	0.74<br>(0.54-0.87)
DLB from EOAD	OF<br>(1.5)	61%<br>(48-72%)	45%<br>(33-58%)	53%<br>(41-65%)	0.52<br>(0.40-0.64)	71%<br>(53-84%)	89%<br>(73-96%)	80%<br>(63-90%)	0.81<br>(0.64-0.91)
DLB from LOAD	CA<br>(0.5)	100%<br>(93-100%)	7%<br>(3-18%)	54%<br>(39-68%)	0.55<br>(0.40-0.69)	86%<br>(65-95%)	57%<br>(36-75%)	71%<br>(50-86%)	0.77<br>(0.55-0.89)
DLB<br>from FTLD-Tau	PA<br>(1.5)	57%<br>(42-71%)	42%<br>(28-57%)	49%<br>(34-64%)	0.46<br>(0.32-0.62)	86%<br>(64-95%)	67%<br>(44-83%)	76%<br>(54-89%)	0.77<br>(0.54-0.90)
DLB<br>from FTLD-TDP43	PA<br>(2.5)	7%<br>(3-18%)	100%<br>(93-100%)	54%<br>(39-68%)	0.50<br>(0.35-0.64)	71%<br>(50-86%)	100%<br>(86-100%)	86%<br>(65-95%)	0.92<br>(0.73-0.98)
DLB<br>from AD+FTLD	CA<br>(0.5)	100%<br>(96-100%)	2%<br>(1-8%)	51%<br>(40-62%)	0.45<br>(0.34-0.56)	86%<br>(71-93%)	47%<br>(32-63%)	67%<br>(50-79%)	0.67<br>(0.51-0.80)
FTLD-Tau<br>from (Younger) Controls	FI<br>(1.5)	92%<br>(80-97%)	94%<br>(83-98%)	93%<br>(82-97%)	0.95<br>(0.85-0.98)	100%<br>(85-100%)	100%<br>(85-100%)	100%<br>(85-100%)	1.0<br>(0.85-1.0)
FTLD-Tau from EOAD	OF<br>(2.5)	58%<br>(45-70%)	82%<br>(70-90%)	70%<br>(57-80%)	0.75<br>(0.62-0.84)	42%<br>(25-60%)	86%<br>(69-94%)	64%<br>(45-79%)	0.58<br>(0.40-0.75)

FTLD-Tau from LOAD	CA (2.5)	50% (35-65%)	96% (86-99%)	73% (58-84%)	0.77 (0.62-0.87)	67% (44-83%)	79% (56-91%)	73% (50-87%)	0.73 (0.50-0.87)
FTLD-Tau from DLB	MTA (1.5)	79% (64-89%)	68% (52-80%)	74% (58-84%)	0.76 (0.61-0.87)	67% (44-83%)	86% (64-95%)	76% (54-89%)	0.77 (0.54-0.90)
FTLD-Tau from FTLD-TDP43	CA (2.5)	50% (35-65%)	82% (67-91%)	66% (50-79%)	0.65 (0.49-0.78)	25% (11-48%)	93% (73-98%)	59% (37-77%)	0.52 (0.32-0.72)
FTLD-Tau from AD+DLB+FTLD-TDP43	CA (2.5)	50% (38-62%)	85% (74-91%)	67% (55-77%)	0.69 (0.57-0.78)	75% (58-86%)	49% (32-65%)	62% (44-76%)	0.61 (0.43-0.76)
FTLD-TDP43 from (Older) Controls	FI (1.5)	93% (82-97%)	94% (84-98%)	93% (83-97%)	0.95 (0.85-0.98)	93% (75-98%)	100% (86-100%)	96% (80-99%)	0.99 (0.84-1.0)
FTLD-TDP43 from EOAD	TA (2.5)	46% (35-59%)	90% (81-95%)	68% (56-78%)	0.73 (0.61-0.82)	93% (79-98%)	47% (31-64%)	70% (52-83%)	0.72 (0.55-0.85)
FTLD-TDP43 from LOAD	OF (1.5)	82% (68-90%)	46% (32-61%)	64% (49-77%)	0.68 (0.53-0.80)	100% (86-100%)	64% (43-81%)	82% (61-93%)	0.81 (0.59-0.92)
FTLD-TDP43 from DLB	TA (2.5)	46% (32-61%)	100% (93-100%)	73% (58-84%)	0.79 (0.64-0.88)	93% (74-98%)	79% (57-90%)	86% (65-95%)	0.92 (0.73-0.98)
FTLD-TDP43 from FTLD-Tau	MTA (2.5)	54% (38-68%)	54% (39-69%)	54% (38-68%)	0.53 (0.38-0.68)	43% (24-64%)	75% (52-89%)	59% (37-77%)	0.52 (0.32-0.72)
FTLD-TDP43 from AD+DLB+FTLD-Tau	TA (2.5)	46% (36-58%)	82% (72-88%)	64% (53-74%)	0.67 (0.55-0.76)	50% (34-66%)	89% (76-96%)	70% (53-82%)	0.71 (0.54-0.83)
