# Supplementary material for: MRI visual rating scales in the diagnosis of dementia: evaluation in 184 post-mortem confirmed cases
Source: Brain. 2016 Mar 1;139(4):1211–25. doi: 10.1093/brain/aww005 (PMC4806219; doi:10.1093/brain/aww005)
Supplement: Supplementary Data [file aww005_supplementary_data.zip › brain-2015-01186-File009.pdf]

	Highest to Lowest Weighted					
	**w1**	**w2**	**w3**	**w4**	**w5**	**w6**
**Early-onset AD vs Young Control**	FI (1.05)	AT (0.48)	MTA (0.46)	PA (0.29)	AC (-0.25)	OF (0.22)
**Late-onset AD vs Old Control**	MTA (0.04)	OF (0.03)	AT (0.02)	PA (-0.01)	AC (0.003)	FI (-0.003)
**DLB vs Old Control**	MTA (0.41)	OF (0.29)	PA (-0.2)	AC (0.19)	FI (-0.17)	AT (0.10)
**FTLD-Tau vs Young Control**	FI (0.15)	MTA (0.13)	OF (0.12)	AT (0.11)	AC (0.11)	PA (0.06)
**FTLD-TDP43 vs Young Control**	MTA (0.87)	FI (0.8)	AT (0.65)	OF (-0.18)	PA (0.11)	AC (0.002)
**Early-onset AD vs Late-onset AD**	PA (1.94)	FI (0.97)	AC (0.87)	MTA (-0.53)	AT (0.36)	OF (-0.34)
**Early-onset AD vs DLB**	AT (0.30)	PA (0.26)	FI (0.23)	AC (-0.09)	MTA (0.08)	OF (0.003)
**Early-onset AD vs FTLD-Tau**	PA (0.13)	AC (-0.12)	AT (-0.11)	FI (-0.09)	OF (-0.08)	MTA (-0.07)
**Early-onset AD vs FTLD-TDP43**	PA (0.18)	OF (-0.17)	AC (-0.09)	AT (-0.08)	MTA (-0.05)	FI (-0.04)
**Late-onset AD vs DLB**	AT (0.64)	AC (-0.62)	MTA (0.18)	FI (-0.07)	OF (0.04)	PA (0.01)
**Late-onset AD vs FTLD-Tau**	AC (-0.03)	AT (-0.03)	FI (-0.03)	OF (-0.03)	MTA (-0.02)	PA (-0.01)
**Late-onset AD vs FTLD-TDP43**	AC (-0.003)	AT (-0.003)	OF (-0.003)	FI (-0.003)	MTA (-0.002)	PA (-0.001)
**DLB vs FTLD-Tau**	AT (-0.03)	AC (-0.03)	MTA (-0.03)	OF (-0.03)	FI (-0.03)	PA (-0.01)
**DLB vs FTLD-TDP43**	AT (-0.62)	PA (0.57)	AC (-0.55)	OF (-0.37)	FI (-0.08)	MTA (-0.08)
**FTLD-Tau vs FTLD-TDP43**	MTA (-0.46)	AC (0.45)	AT (0.39)	PA (-0.21)	OF (0.02)	FI (0.02)
