# Supplementary material for: MRI visual rating scales in the diagnosis of dementia: evaluation in 184 post-mortem confirmed cases
Source: Brain. 2016 Mar 1;139(4):1211–25. doi: 10.1093/brain/aww005 (PMC4806219; doi:10.1093/brain/aww005)
Supplement: Supplementary Data [file aww005_supplementary_data.zip › brain-2015-01186-File010.pdf]

T	Amsterdam			London			Newcastle		
	**1T**	**1.5T**	**3T**	**1T**	**1.5T**	**3T**	**1T**	**1.5T**	**3T**
**AD**	7	1	1	0	77	6	1	8	0
**Control**	0	0	0	0	55	18	0	0	0
**DLB**	2	0	2	0	6	0	3	15	0
**FTLD**	8	1	1	0	40	4	0	1	0
**Total**	**23**			**206**			**28**		
