# Supplementary figures and images for: MRI visual rating scales in the diagnosis of dementia: evaluation in 184 post-mortem confirmed cases
Source: Brain. 2016 Mar 1;139(4):1211–25. doi: 10.1093/brain/aww005 (PMC4806219; doi:10.1093/brain/aww005)

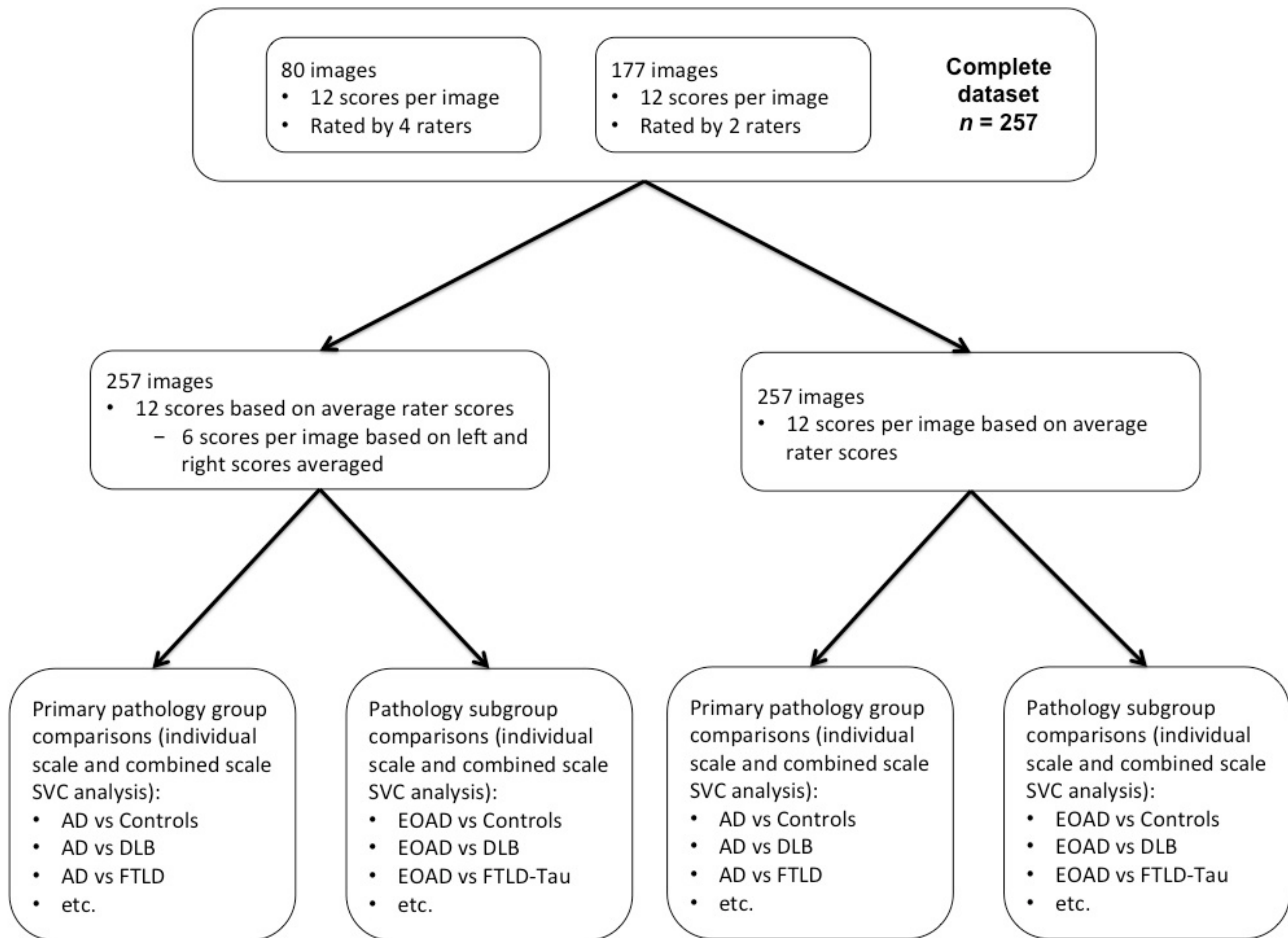

Supplement: Supplementary Data [file aww005_supplementary_data.zip › brain-2015-01186-File011.pdf]

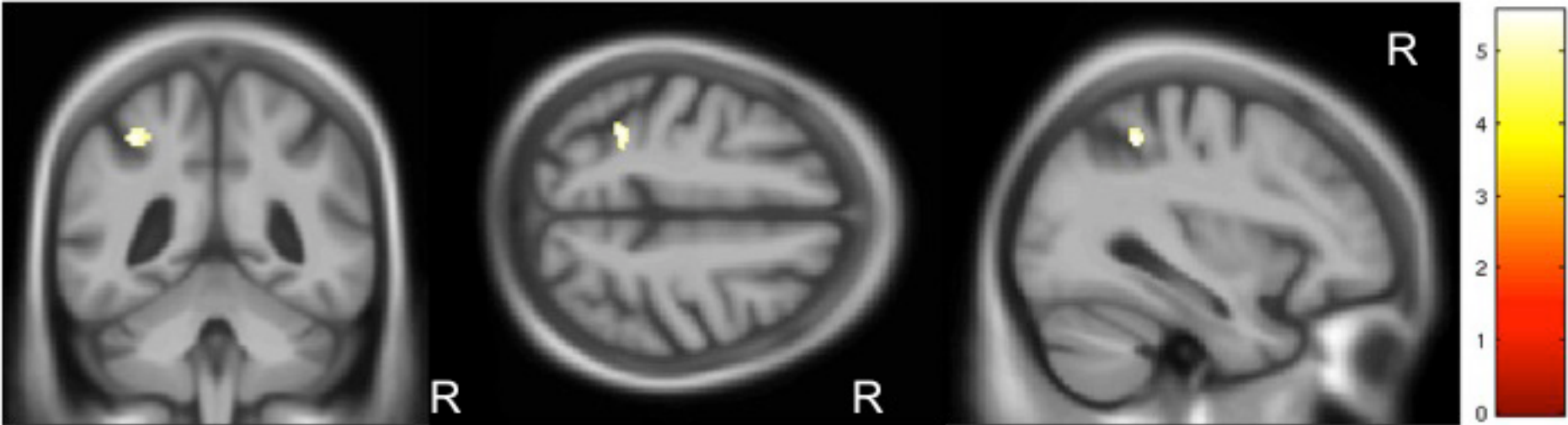

Supplement: Supplementary Data [file aww005_supplementary_data.zip › brain-2015-01186-File012.pdf]

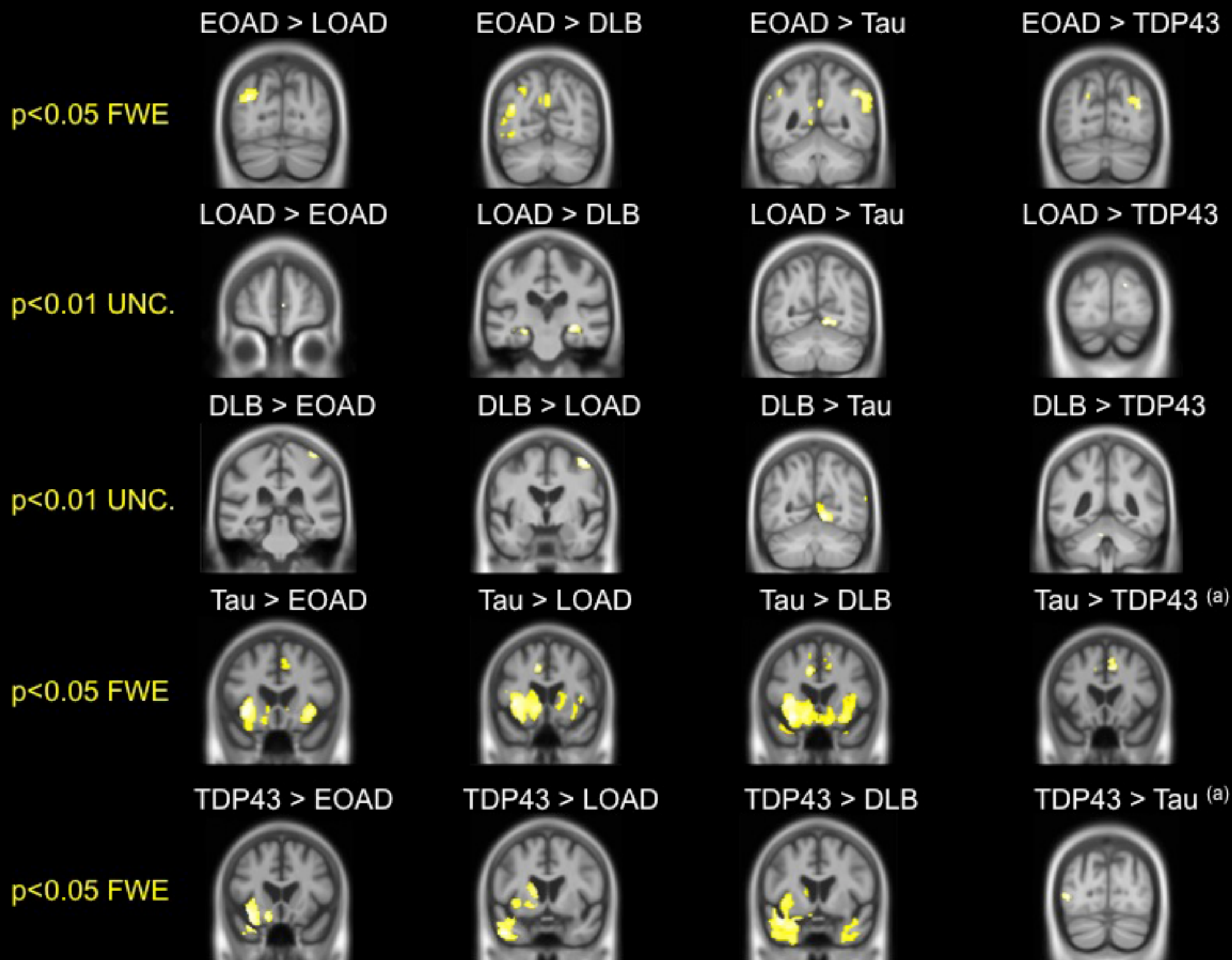

Supplement: Supplementary Data [file aww005_supplementary_data.zip › brain-2015-01186-File015.pdf]

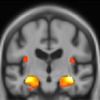

Supplement: Supplementary Data [file aww005_supplementary_data.zip › brain-2015-01186-File017.png]
