# Supplementary material for: MRI visual rating scales in the diagnosis of dementia: evaluation in 184 post-mortem confirmed cases
Source: Brain. 2016 Mar 1;139(4):1211–25. doi: 10.1093/brain/aww005 (PMC4806219; doi:10.1093/brain/aww005)
Supplement: Supplementary Data [file brain-2015-01186-File014.pdf › brain-2015-01186-File014.pdf]

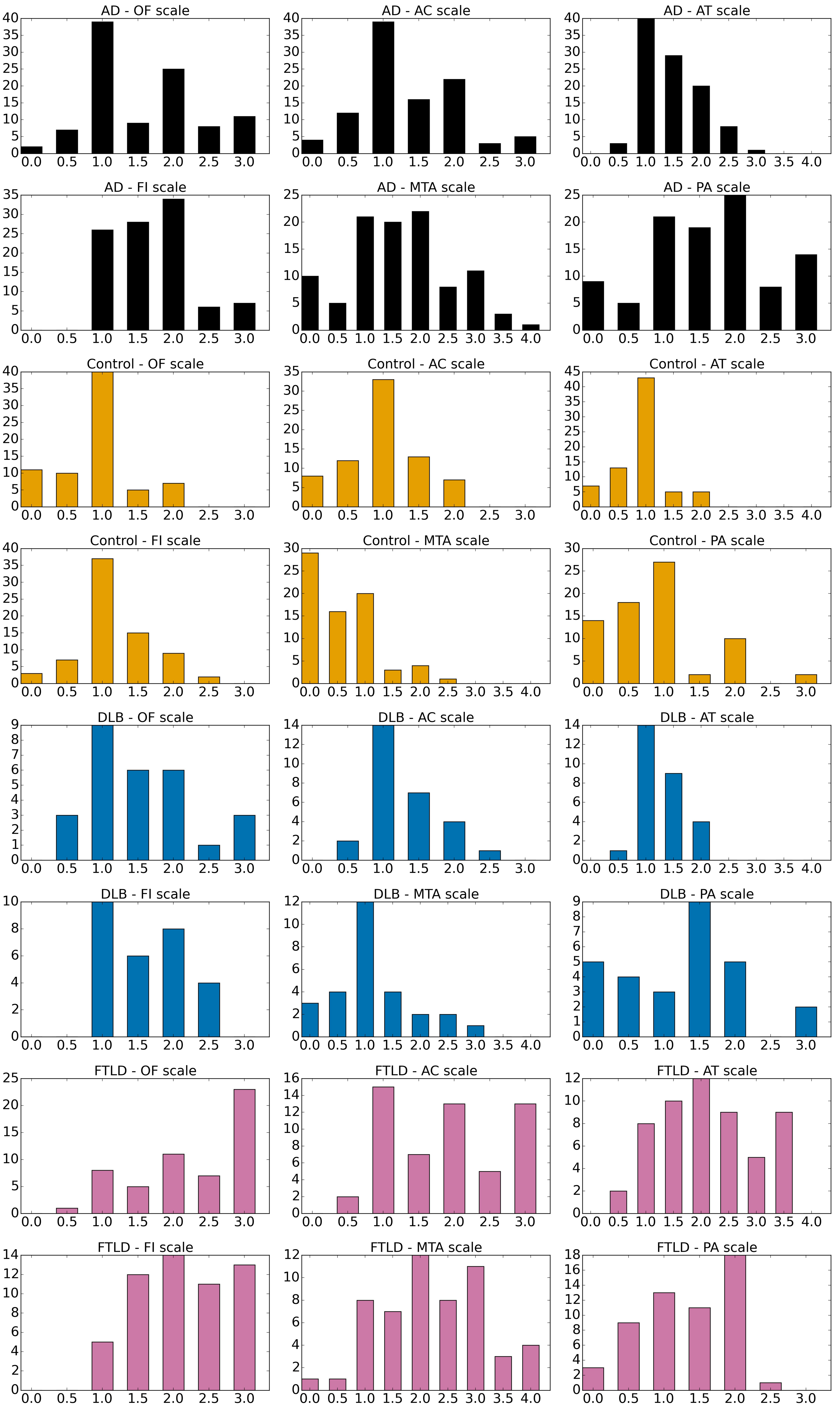
AD - OF scale
AD - AC scale
AD - AT scale
AD - FI scale
AD - MTA scale
AD - PA scale
Control - OF scale
Control - AC scale
Control - AT scale
Control - FI scale
Control - MTA scale
Control - PA scale
DLB - OF scale
DLB - AC scale
DLB - AT scale
DLB - FI scale
DLB - MTA scale
DLB - PA scale
FTLD - OF scale
FTLD - AC scale
FTLD - AT scale
FTLD - FI scale
FTLD - MTA scale
FTLD - PA scale
